# Challenges to climate change adaptation in coastal small towns: Examples from Ghana, Uruguay, Finland, Denmark, and Alaska

**DOI:** 10.1016/j.ocecoaman.2021.105787

**Published:** 2021-10-15

**Authors:** James M. Fitton, Kwasi Appeaning Addo, Philip-Neri Jayson-Quashigah, Gustavo J. Nagy, Ofelia Gutiérrez, Daniel Panario, Inti Carro, Leo Seijo, Carolina Segura, José E. Verocai, Samrit Luoma, Johannes Klein, Ting-Ting Zhang, Jeff Birchall, Peter Stempel

**Affiliations:** a Department of Planning, Aalborg University, Denmark; b MaREI, Environmental Research Institute, University College Cork, Ireland; c Institute for Environment and Sanitation Studies (IESS), University of Ghana, Ghana; d IECA, Facultad de Ciencias, Universidad de la República, Uruguay; e Dirección Nacional de Cambio Climático, Ministerio de Ambiente, Uruguay; f Programa de Desarrollo y Gestión Subnacional, OPP, Presidencia de la República, Uruguay; g Dirección Nacional de Biodiversidad y Servicios Ecosistémicos, Ministerio de Ambiente, Uruguay; h Geological Survey of Finland, Finland; i School of Urban and Regional Planning, Department of Earth and Atmospheric Sciences, University of Alberta, Edmonton, Canada; j Department of Landscape Architecture, The Pennsylvania State University, Pennsylvania, USA

**Keywords:** Coastal hazards, Climate change, Small settlements, Adaptation

## Abstract

The ability of a coastal settlement to adapt to climate change is largely dependent upon access to a range of resources, which many coastal towns and small cities lack. Coastal small towns of less than 10,000 are therefore at a significant disadvantage compared to larger settlements when it comes to adaptation. One way to begin to overcome this disadvantage is to compare coastal small towns in order to identify efficiencies and support knowledge sharing. In this article we present and analyse five case studies of coastal small towns: Fuvemeh, Ghana; Kiyú, Uruguay; Hanko, Finland; Lemvig, Denmark; and Nome, Alaska, USA. A number of key outcomes and lessons were identified which highlights the need for a formal network of international coastal small towns to encourage and develop knowledge sharing practices going forward. A further lesson is the importance of using a range of indicators in order to establish the regional/national importance of a town. Basing this solely on population size can result in an erroneous interpretation of the significance (and therefore adaptive capacity) of a coastal small town. Finally, despite many barriers to adaptation in coastal small towns, being small offers some potential advantages, such as the possibility of being able to form a community consensus more easily, using 3D visualisations for adaptation planning, and having managed realignment as a realistic management option. It is imperative that climate change resilience in coastal small towns is increased by focussing on overcoming barriers and developing appropriate adaptation approaches by governments, non-governmental organisations, business, and researchers.

## Highlights

• Five case studies are used to gain insight into adaptation in coastal small towns.
• Adaptation barriers and possible adaptation approaches are explored.
• Barriers include narrow economies, limited data and the scale of decision making.
• Forming community consensus and managed realignment may be viable in small towns.
• More focus is required on small coastal towns to address the barriers to adaptation.

## 1 Introduction

The coast is a desirable place to live, with coastal population densities three times the global average (Small and Nicholls, 2003). It is estimated that 625 million people live within the low elevation coastal zone (the area below 10 m elevation) (Neumann et al., 2015). However, there are a number of coastal hazards, such as coastal erosion, storm surges and flooding, which can impact upon coastal settlements. Rising sea-levels and changes in the frequency and severity of storms due to climate change will likely exacerbate these hazards (Masselink and Russell, 2013; Neumann et al., 2015; Vitousek et al., 2017; Werner et al., 2012). With an in increase in the severity of coastal hazards, and the expected growth within coastal settlements (Neumann et al., 2015), it is imperative that coastal settlements adapt to both current and expected coastal hazards to increase resilience and reduce risk to climate change.

The ability of a coastal settlement to adapt is largely dependent upon access to climate, engineering, and management knowledge; access to accurate and reliable local scale climate data; access to financial resources; local stakeholders’ participation; and political consensus (Berman et al., 2020; Gibbs, 2015; Harman et al., 2015; Major et al., 2018; Rosenzweig et al., 2018). Major and Juhola (2016) highlight that many coastal towns and small cities lack many of these resources, and therefore larger and wealthier coastal cities have a greater ability to adapt to climate change. Small settlements are therefore thought to be at a significant disadvantage when it comes to adaptation (Bell and Jayne, 2009; Birchall and Bonnett, 2019, 2020; Birkmann et al., 2016; Hamin et al., 2014; Paterson et al., 2017; Pauleit et al., 2015). However, this assumption has not been fully explored at the local level, and although disadvantages are prevalent, there may be a number of factors associated with being small that promote adaptation.

This article is part of 'The Unusual Suspects in Climate Change Adaptation – Small Coastal Cities and Towns' Special Issue. This article focuses on coastal small towns (less than 10,000 people) that are situated on the mainland. Coastal adaptation in five small towns from around the world are examined in order to inform an analysis of the key problems and hazards, barriers to adaptation, and knowledge gaps. These five cases represent a diverse climatic, management, environmental, and socio-economic settings. In the following section, the cases used within this paper will be briefly introduced. The management challenges within these small towns will be summarised in section 3, with examples of implemented adaptation outlined in section 4. Finally, section 5 will present some of the potential advantages of being small and highlight some of the potential adaptation approaches that are highly applicable to coastal small towns.

## 2 Case studies

**Fig. 1:**
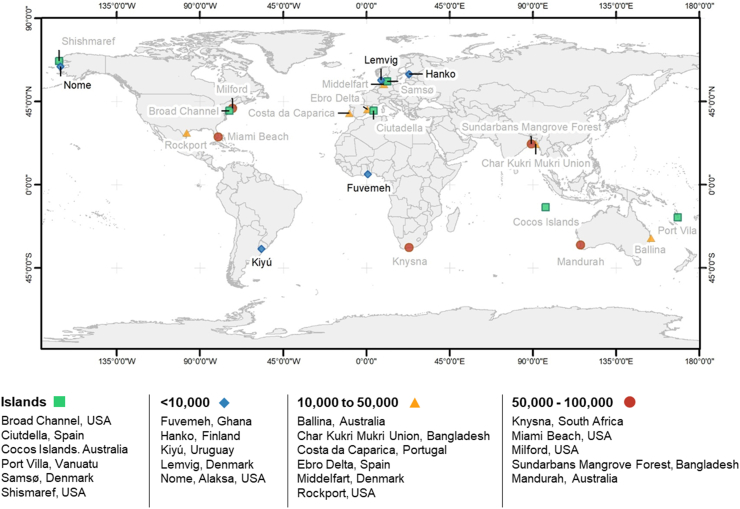
Map of the case study locations within the complete Special Issue. This article analyses coastal adaptation within the small town (less than 10,000 people) case studies. See Lehmann et al. (2021a) for further information on the Special Issue.

The cases included here are derived from a call for cases to be included within the Special Issue. The approach used to develop the Special Issue is discussed further in Lehmann et al. (2021a). In total, 22 cases are focussed upon in the Special Issue (Fig. 1), five of which were classified as small (less than 10,000 people) and the focus of this article. The five cases (Table 1) differ in terms of the hazards they face, the economic context in which they are located, and the extent of management/adaptation that has already been implemented.

**Table 1:** Summary of the coastal small town case studies, which span a range of geographic settings, hazards, and impacts, used to generate the insights about climate change adaptation. For further information and sources on each case refer to the supplementary material of this article.

	Fuvemeh, Ghana	Hanko, Finland	Kiyú, Uruguay	Lemvig, Denmark	Nome, Alaska, USA
**Description**	Local fishing village	Town with tourist visits during the summer months	Town with tourist visits during the weekends and summer months	Town with a multi-functional sea wall	Regionally significant town based in the sub-arctic
**Population**	1500	8517	500	6936	3598
**Built Infrastructure**	Approximately 200 buildings sparsely distributed in the community; basic school	Ports; transport infrastructure; water intake wells; waste water treatment plants; health centre; airport; highway; railway.	Few hundred buildings, mostly summer houses; basic transport infrastructure	Harbour; key road; residential and commercial property	1503 housing units; roads; port and harbour; airport; regional hospital
**Human Development Index**	0.592	0.920	0.804	0.929	0.924
**Hazard Summary**	Coastal erosion and storm surge flooding	Decrease in groundwater volume and quality	Coastal erosion and storm surge flooding	Storm surge flooding	Permafrost thaw damaging assets and sea ice decline increasing erosion and flooding risk
**Sea Level Rise**	Currently 3.1 mm yr^−1^ and expected to accelerate significantly	Currently 1.5 mm yr^−1^ with sea level expected to rise by 0.51 m by 2100	1–2 mm yr^−1^ over the past five decades	Expected to rise between 0.3 and 0.7 m by 2100	Limited empirical evidence, but very little relative sea level rise expected
**Coastal Erosion**	Current erosion rate of 7.24 m yr^−1^	Erosion on beaches by winds and waves	Erosion rate of 1 m yr^−1^ over past 1000 years	Negligible -harbour is mainly reclaimed land and protected by hard defences	Significant coastal erosion

For each of the five cases, researchers with experience of the location have completed a typology and a short case study narrative that summarises the current situation within the small town (See Lehmann et al., 2021a, Lehmann et al., 2021b for further details on the methodological approach). Both the typology and the narratives were used to generate insights about climate adaptation. The full typologies and narratives are included within the supplementary materials of this article. A short summary the narrative of each of the cases is included below. Due to the cases emanating from a range of countries and contexts, the information provided within these narratives and typologies is not uniform as they focus on particular local issues. However, they can be developed further with time, as they constitute a resource that can be utilised within future research.

### 2.1 Fuvemeh, Ghana

Fuvemeh is a small coastal community of about 1500 people (Ghana Statistical Service, 2012), located on a narrow stretch of sandbar positioned between the Volta River and the Gulf of Guinea, on the eastern side of the Volta Estuary. In 1963, the Akosomba Dam was constructed on the Volta River which significantly reduced sediment transported to the coast. As a result, the coastal town of Ada, on the western side of the Volta River started to experience coastal erosion. The reaction to this was to install groynes along the beach of Ada in 2013–2017. This further reduced the sediment reaching Fuvemeh, resulting in increased erosion rates. Fuvemeh was once a vibrant fishing community but is gradually being lost due to increasing coastal erosion and flooding. Although there are issues with regards to the coastal management approach, these problems will be exacerbated further by sea level rise.

### 2.2 Hanko, Finland

The town of Hanko is located on the Hanko Cape on the Baltic coast in southern Finland. The population of Hanko was 8517 at the end of 2017 (City of Hanko, 2018) and according to projections, will decrease to 7380 by 2040 (City of Hanko, 2017a). Hanko has a long history dating back to the 13th century, and has an economy based on services and industry. Hanko is a popular summer resort due to the local culture, the unique nature, and the long sandy beaches. Consequently, the arrival of holiday homeowners and tourists, increases the population considerably during the summer. Hanko is relatively low-lying and has long sandy beaches meaning rising sea levels due to climate change will exacerbate storm surges and increase flood-risk and erosion along the coast. This could also impact up on freshwater quality and availability due to seawater intrusion in flood-prone areas.

### 2.3 Kiyú, Uruguay

Kiyú is located in on Uruguay's southern coast, along the Rio de la Plata's estuarine coast. The town has 500 inhabitants (as of 2018), reaching 3000 to 4000 due to tourism during the summer months (January–February), peaking at ≥10,000 people during weekends. A moderate increase in inhabitants is expected in the near future.

Two successive extreme storm surges in September and October 2012 impacted Kiyú Beach. The coastline, characterised by erosive bluffs, sandy beaches and dunes, is vulnerable to storm surges, and flooding associated with riverine floods (triggered by El Niño events). The storm surges eroded the sandy beach, dune, and bluffs, while fallen trees broke the bluff; a touristic site and coastal roads were also damaged. Sea-level due to climate change will increase the likelihood of more damaging storm surges.

### 2.4 Lemvig, Denmark

Lemvig is a small municipality on the west coast, in the Midtjylland region of Denmark. Lemvig has a population of 6936. The municipality has a negative population growth and more than 50% of the residents are aged over 40. While the overall population growth is negative, the population of those aged 65 and above is expected to increase (Lemvig Kommune, 2017). The biggest concern for Lemvig is flooding: storm surges, overflow of sewers in cloud burst events, flooding from increased storm intensity, groundwater flooding, and flooding along streams (Lemvig Kommune, 2014). Flooding was an issue already in Lemvig, but climate change is likely to exacerbate this further. For example, the rise in sea level due to climate change will reduce the return period of storm surge events, with the 10-year storm surge event elevation rising from 166 cm, to 260 cm by 2120 (COWI, 2017).

### 2.5 Nome, Alaska, USA

Nome is a small community situated on the southern coast of the Seward Peninsula, in northwest Alaska, USA. Nome is remote from major urban centres in the interior, such as Anchorage and Fairbanks, with access limited to sea and air (Anchorage: 870km; Fairbanks: 840km) (City of Nome, 2012). Local access to outlying communities is restricted to seasonal roads. Incorporated in 1901, Nome was once a bustling gold mining town of over 20000 people (City of Nome, 2017). Since then, Nome's population has decreased significantly, primarily as a result of a decline in the gold industry, disease outbreak (i.e. Spanish Flu) and war. With a current population of 3,598, Nome remains the largest community in the region, and serves as a hub for the provision of health and education, and commercial and industrial activities. However, rising temperatures due to climate change have resulted in permafrost thaw, causing building subsidence, utilities failures, and road damage; and, sea ice decline, causing larger wave fetch and reduced wave energy dissipation, contributing towards greater flooding and erosion risk.

## 3 Management challenges

The case studies above face similar combinations of coastal hazards, however the impacts and the management (both reactive and proactive) within these towns is not the same. Within this section the case studies will be explored to identify some of the key factors that should be considered when developing management and adaptation strategies. These relate to the local economy, data and information access, and decision making.

### 3.1 Local economy

In a number of the case studies the local economy is based upon a narrow range of industries, and in some cases only a single industry. As a result, if small town is impacted by a coastal hazard there may be significant short- and long-term impacts on the town. Fuvemeh, which was a thriving fishing village, has lost properties due to erosion, and the day to day life of the community has been severely disrupted, hindering the ability of the residents to undertake fishing activities, resulting in lost food and income for the local community. Kiyú also has potential problems due to the town being highly reliant upon tourism as its main source of revenue. The other localities presented here are not as reliant upon a single industry, however, any disruption of economic activity has the potential to have a proportionally large effect on the towns.

The example of Fuvemeh and Kiyú highlight the possibility that if the local economy and infrastructure of a town is continually being harmed and/or irreparably damaged, the motivation and ability to take management action is weakened. Consequently, a town may reach a ‘tipping point’ where it is never able to recover (Franck, 2009). Small towns are more likely to reach the tipping point where the costs of intervention and adaptation greatly outweigh the benefits. A tipping point in larger towns and cities will also exist, but it will take longer or more severe hazard events to reach this threshold.

The case of Hanko demonstrates how the local economy can also exacerbate the impacts of climate change on a small town. Hanko residents and industry rely upon a shallow ground water aquifer for their water needs. The expected relative rise in sea level (0.51 m) in the area would cause some parts of the Hanko aquifer to be below sea level, affecting groundwater volume and quality due to saline intrusion. This problem may be further exacerbated during the summer months when Hanko receives a high volume of tourists and visits by summer home owners. This seasonal rise in groundwater use during the drier months of the year may result in lowering of the groundwater level, and increasing the risk of saline intrusion. Consequently, drinking water quality will reduce and there would be potential water shortage during the summer.

### 3.2 Data and information access

A key barrier to adaptation highlighted by Major and Juhola (2016) is access to information at a scale useable to a small town. In the case studies used here, data and information has been drawn from numerous IPCC, continental/regional, and national climate change reports or analysis to determine the degree of climate change locally. While it is important to have data and information on the global and regional impacts of climate change, there is a lack of specific reports or analysis that assess the implications of climate change at the local scale (Birkmann et al., 2016).

Where local scale data or information is utilised within the five case study typologies the sources for the local climate change impacts are generally from academic research. Given the difficultly to obtain local information; this demonstrates the advantage of having an active research community that can potentially contribute towards filling knowledge gaps within small towns. However, not every small town can be subject to academic research as universities may not be within vicinity, lack the relevant research focus, or have the funds to support such research. Therefore, it cannot be seen as a viable option for improving local knowledge within all coastal small towns.

Lemvig has the benefit of a national coastal authority (*Kystdirektoratet*) being based in the town, whose responsibilities include collecting and analysing coastal dynamic data, as well as making coastal management recommendations to government. Both Lemvig and Hanko have been part of European Union (EU) research projects. Lemvig is current member of the EU Life project Coast to Coast Climate Challenge (C2C CC, n.d.) and Hanko was part of the BaltCICA Project: Climate Change: Impacts, Costs and Adaptation in the Baltic Sea Region (Schmidt-Thomé and Klein, 2013). Both of these projects include contributing and sharing knowledge across a network of project partners. In the case of the BaltCICA this network consists of international partners, whereas the EU Life Coast to Coast project includes partners across municipalities within Denmark. Projects such as these are an efficient way to produce knowledge, but also establish collaborative networks that have potential to extend beyond the life of the initial project. Small towns that are part of these networks then have the opportunity to acquire more data, expertise, and knowledge than they would usually be able to access.

### 3.3 Decision making

Access to local scale data and information is important, but how it is used is equally critical. How decisions are made and by who is key, with a number of different forms of decision-making and governance represented within the cases presented here. It is worth highlighting Nome, as although small, its influence greatly exceeds the boundaries of the town.

Nome has a population of 3,598, yet this is the largest settlement for a significant distance, and therefore acts as a hub for much of northwest Alaska. Consequently, situated within Nome is; a substantial airport that receives approximately 77 operations per day; a port that is the sole provider of moorage and services in the region; the main hospital for the Norton Sound Health Corporation, which covers all communities with the Bearing Strait region (an area of 114,000 km^2^); four schools; and a community campus of the University of Alaska Fairbanks (City of Nome, 2012). Hence, despite its small population, Nome is highly important to the region, and decisions and response to coastal and climate hazards in the town will affect a much greater number of people than just those within the immediate vicinity. This supports the observation of Rondinelli that functions within towns and small cities “seem to increase in diversity and complexity not only with density of settlements but also with their accessibility to hinterland populations'’ (Rondinelli, 1983, p. 386). This has two main implications; firstly, Nome has a responsibility to act in a manner that benefits both its inhabitants and the wider region; secondly, as Nome has a wide range of stakeholders, support from the state or national government, is potentially more readily available. Nome therefore does not necessarily have the same degree of barriers to adaptation as initially outlined by Major and Juhola (2016).

However, this does not equate to Nome leading on climate change adaptation. A number of reports conducted by the City of Nome establish some of the potential impacts of climate change, and a number of decision makers accept the impacts of climate change on the community. Yet, Birchall and Bonnett (2020) note that city officials characterise their efforts as reactionary, rather than anticipatory, with climate change adaptation given a low priority within the community's strategic planning documents and zoning policy. Therefore, it is not only a case of improving access to data and information, but also supporting decision makers in using this resource (Birchall and Bonnett, 2019).

Small towns that are more isolated and/or have few stakeholders will also struggle to adapt and be further negatively impacted by neighbouring communities if an integrated approach to the coast is not adhered to. This is demonstrated by Fuvemeh, which was impacted by reduced sediment load from the Volta River due to construction of the Akosomba Dam. The coastal town of Ada, on the western side of the Volta experienced coastal erosion as a result. To address this the beach was nourished and groynes were installed in 2013–2017. The construction of the groynes, caused the Fuvemeh coast to change from accretional to erosional. In the modelling and design of the Ada coastal protection scheme outlined by Bollen et al. (2011) and Bolle et al. (2015), the impact of the scheme on Fuvemeh was not mentioned, and therefore not taken into consideration in the design of the protection. Ada, while still a small town (approximately 5700 people in 2010 (Ghana Statistical Service, 2014), is larger than Fuvemeh, and consequently the interest of the smaller town were not a high priority for the decision makers in Ada.

An approach to improve the coastal management decision-making process was used in Kiyú by engaging with local stakeholders. The participatory assessments were held with different groups of stakeholders, managers, and non-governmental organisation (NGO) members to determine vulnerability and risk perception, as well as barriers and opportunities to implement adaptation (Carro et al., 2018). While a decision to reprofile the beach had already been agreed, these consultations helped to prioritise further actions from a series of pre-selected adaptation measures. This kind of participation in local governance and monitoring legitimise management actions and facilitates the reduction of the perception of vulnerability, while also serving as an input for Scenario Planning for new adaptation plans (Nagy et al, 2014, 2017).

Further examples of working with a wider network of stakeholders has been demonstrated in Hanko with projects either planned or implemented. These include cooperating with neighbouring towns on the water supply and management issues and detailed investigations of hydrogeology of the aquifers for better groundwater protection plans and management to ensure there is sufficient clean water for the communities in the future.

## 4 Examples of management

A common theme through this Special Issue is that while there are many examples of research and planning, few cases demonstrate actual intervention. However, three of the five cases presented here have implemented some form of management to reduce the impacts of climate change.

To overcome the potential drinking water issue in Hanko, a connection to the neighbouring town of Raasepori has been implemented to allow sharing of water (City of Hanko, 2017b; Hanko Water Department, pers. communication, 2016). This removes the pressure on the local aquifer, especially during the summer months. This measure was not directly on the coast; however, it has contributed to solving a coastal hazard, demonstrating the value in small communities working with other nearby towns/cities.

Within Kiyú a number of low-cost measures were implemented that were focussed on ecosystem-based adaptation. The measures included sand capture fencing to increase beach height and volume (see Carro et al. (2018) for a summary of the implemented measures). As a result of the fencing the beach profiles between 2013 and 2018 showed an increase in the average height (≥1 m) and volume (≥46%) of sand, a well-developed high water level berm, and a more massive vegetated dune. Consequently, none of the six storm surges, including surges similar to the ones of 2012 that have occurred since the implementation, have put the coastal infrastructure at risk. This relatively cheap and simple approach demonstrates that ecosystem-based adaptation can be very effective. It also serves as an example of how to engage with the local community so that support for such approaches can be achieved, and therefore avoid hard-engineering solutions, such as sea walls, which have the potential to create further problems (Kraus and McDougal, 1996; Kraus, 1988).

Lemvig is located on the Limfjord and has experienced flooding due to storm surges. Consequently, the Midtjylland Municipality chose to install a sea wall along the waterfront, as well as redesign the waterfront layout in in 2012–13. The sea wall was designed to be multifunctional, and has a number of gates within it, which are open during normal conditions and allow the public to walk around and utilise the area to sit, as well as access a children's play area (Faragò et al., 2018). During adverse weather the gates in the wall are closed, and the wall acts like a traditional barrier. The wall is designed to prevent flooding 2.1 m above sea level, and since its construction a number of storm surges have occurred, with the seawall successfully preventing flooding in the town. This limits the life of the sea wall, as with sea level rise, storm surges and wave overtopping are expected to regularly exceed this height. Initially, the sea wall was expected to give protection for approximately 25 years, with the sea wall being redesigned or upgraded in the future (Østergaard, 2015). The sea wall provides benefits beyond just coastal protection, and is worth highlighting this as a possible route to creating cost efficiencies in the implementation of coastal adaptation is by implementing actions with additional co-benefits.

Due to the limit on resources, especially financial, in coastal small towns, there is a clear benefit in making cost effective decision and implementing adaptation that adds wider/additional benefits to the local population. There is potentially more support for the local community if adaptation is implemented in this manner, particularly when it comes to softer or controversial forms of management, such as managed realignment.

## 5 Potential coastal adaptation approaches

While there are clear negatives associated with being a small town, it can provide some advantages over larger coastal settlements. Firstly, within a small town it is potentially easier to achieve community consensus on future management approaches, as shown by stakeholder engagement within Kiyú. Within larger towns and cities, there may be more conflicting views on the management approaches to take, leaving decision makers with no clear direction. A smaller community therefore may be more open to the use of softer forms of intervention, as these can be relatively inexpensive to implement and the potential benefits to the wider community, not just one particular set of stakeholders, can be more readily seen. Small towns could potentially be the ideal place to explore the use of more experimental or novel forms of coastal management, which can be expanded to larger towns and cities as appropriate.

A seldom used approach to managing the coast is managed realignment. This involves relocating assets at the coast to areas that are no longer at risk. It is potentially a very effective long-term approach as it essentially removes assets from harm in a sustainable manner. However, it is difficult to implement as there are many environment, economic, cultural, and political factors to consider. For example, in the cases explored here, parts or all the assets in Fuvemeh could be moved, however, the economic costs associated with implementing this may be prohibitive in the local context. These factors grow considerably with an increasing settlement size, and there is a point where a settlement is too large to consider managed realignment as a viable option. However, as mentioned above, building a community consensus on future management approach is potentially easier in small towns. This could be harnessed to garner support for a managed realignment approach within these coastal small town communities and secure a more viable long-term future. Again, with increased uses of these approaches, larger towns and settlements can learn lessons, and although moving whole cities might not be appropriate, elements of the approach could be utilised in regions of the larger settlements.

Using 3D visualisations can be an effective approach to inform the community about climate change impacts and management options which are increasing being used (Schroth et al., 2014; Sheppard, 2005; Wang et al., 2018). As outlined in 3.2, obtaining the data to support the creation of visualisations in small towns is problematic. However, small towns may require more modest amounts of data to create visualisations and smaller geographic scale could, in some cases, make it easier to create visualisations. This combined with the increasing availability of no cost tools such as Google Earth suggest that visualization could play a larger role in adaptation of small towns if modest amounts of technical skill are available. Experts have raised concerns about the creation of misleading visualisations by non-experts (Sheppard and Cizek, 2009). Issues arise from improper downscaling of data, resolution and vertical accuracy of available data, combination of data of incompatible types, and the characterisation of uncertainty (Kostelnick et al., 2013; Liu and Palen, 2010).

These issues notwithstanding, visualisations need not be optimal to be effective. Visualisations used in community settings can act as shared points of reference that foster interchange among diverse stakeholders (Star, 2010). In these circumstances, shared understandings of risk that are distinct from the scenarios presented are negotiated among the stakeholders (Becker, 2017). The efficacy of these processes depends on disclosing assumptions, engaging stakeholders in scenario development and responses (Sheppard et al., 2013). Thus, emphasis should be brought to the holistic management of engagement processes.

## 6 Conclusion

Due to the limited resources available to small towns, coastal adaptation to climate change is hindered. Therefore, it is essential that the status and implementation of adaptation in small towns is more widely shared so that knowledge and expertise can be more efficiently acquired by communities that require it. Even though only a small number of cases were included here, similarities have been identified which shows that coastal small towns can learn a lot about adaptation from each other. This further highlights the need for a formal network of international coastal small towns to encourage and develop knowledge sharing practices going forward. However, networking and collaborating at the local level, and following an integrated coastal management approach, is just as vital.

In order to support the formation and development of this network it is key to have a data collection protocol that allows connections between small towns to be identified easily. In the trial of the typology within this Special Issue (Lehmann et al., 2021b), this has proved to an important resource. A key outcome is that population size can be used as an approximate estimate of the national/regional importance of a coastal small town, however, there are some cases where this is not suitable. The example of Nome exemplifies this as it is a small town, but it's significance far exceeds what the population size may suggest. This confirms the benefit of using a typology with diverse indicators that attempts to capture a range of factors that should be taken into account when considering coastal adaptation. A varied typology therefore provides the ability to draw strong parallels between similar coastal towns and establish worthwhile and potentially fruitful relationships for adaptation knowledge sharing.

Despite many barriers to adaptation in coastal small towns, there are indications from the cases that some coastal small towns may be able to implement adaptation approaches that may be unsuitable in larger coastal towns. The possibility of being able to form a community consensus, using 3D visualisations, and having managed realignment as a realistic option are not always possible within larger settlements. There is a requirement for a consolidated effort by governments, non-governmental organisations, business, and researchers to explore these potential opportunities and increase resilience of coastal small towns to the impacts of climate change.

## Declaration of competing interest

There are no conflict of interest with regards to the article entitled “Challenges to Climate Change Adaptation in Coastal Small Towns: Examples from Ghana, Uruguay, Finland, Denmark, and Alaska”.
