# Supplementary material for: Challenges to climate change adaptation in coastal small towns: Examples from Ghana, Uruguay, Finland, Denmark, and Alaska
Source: Ocean Coast Manag. 2021 Oct 15;212:105787. doi: 10.1016/j.ocecoaman.2021.105787 (PMC10644629; doi:10.1016/j.ocecoaman.2021.105787)
Supplement: Multimedia component 1 [file mmc1.pdf]

Vulnerability of coastal community to climate change in the Volta Delta, Ghana

Kwasi Appeaning Addo[1] and Philip-Neri Jayson-Quashigah[1]

[1]Institute for Environment and Sanitation Studies (IESS), University of Ghana, Ghana

Abstract

Coastal hazards such as flooding and erosion, as a result of climate change induced sea level rise and strong ocean wave action along the Volta Delta in Ghana, has increased the risk levels in vulnerable communities in the Volta delta region significantly. These hazards interact with increasing human activities in the coastal zone to drive changes in the shoreline fronting the delta. This complex interaction has resulted in the destruction of sources of livelihood, threatened life and property, displaced households and increased poverty. This study assessed the changing trends in the shoreline fronting the Volta delta and how it has contributed to increased flooding incidence in Fuvemeh, a vulnerable coastal community in the Volta delta. The study revealed that between 2005 and 2017, the shoreline fronting Fuvemeh community has moved several meters inland (over 100 m along some transects) in some areas, while in other areas land has been gained. It also emerged that over 77 houses have been destroyed by erosion and flooding, which resulted in the displacement of over 300 inhabitants in Fuvemeh. It was also discovered that a sandbar on the eastern side of the estuary breached resulting in the formation new features, which resulted in changes in the position of the Volta River mouth and the impact of the swell waves. The study has revealed the impact of climate change on the vulnerable Fuvemeh community and demonstrated the need to develop effective adaptation approaches to increase the resilience of the inhabitants in the community.

1.0 Introduction

Coastal erosion dominates majority of the coastline of Ghana and threatens coastal infrastructures significantly (Angnuureng et al., 2013; Appeaning Addo et al., 2008; Boateng, 2012b; Jayson-Quashigah et al., 2013; Ly, 1980; Wiafe et al., 2013). Coastal erosion and flooding have displaced households and destroyed sources of livelihoods in some communities in the Volta delta (Appeaning Addo et al., 2018; Boateng, 2012a). One of such delta communities is Fuvemeh, a small coastal community of about 1500 people (Ghana Statistical Service, 2012). Fuvemeh is located between longitudes 0.6898° to 0.7107° E and latitude 5.7706° to 5.7754° N on the eastern side of the Volta Estuary (Figure 1). It is on a narrow stretch of sandbar sandwiched

*Corresponding Author: KAppeaningAddo@ug.edu.gh

between the Volta River and the Gulf of Guinea. Wave approaching this area have a significant wave height of about 1.4m and the dominant wave direction is south-south-west (SSW) (Angnuureng et al., 2013). Tidal range is about 1 m and therefore tidal current has minimum influence on the shoreline morphology (Angnuureng et al., 2013). The community, which was once a vibrant fishing community, is being wiped away gradually because of increasing coastal erosion and flooding due to energetic swell waves and storm surge. High rates of erosion in Ada community on the western side of the Volta estuary resulted in the Ada sea defense project using groynes in 2013 (Bollen et al., 2011).

2.0 Approach

The current situation at Fuvemeh was assessed by analyzing historical images (2005 to 2017) obtained from the Ghana Survey Department and the Digital globe foundation. The images (Table 1) were pre-processed and the shoreline positions as well as buildings were extracted from them. Household population was estimated using data from the Ghana Statistical services and the DECCMA Project household surveys in 2017 (Abu et al., 2017).

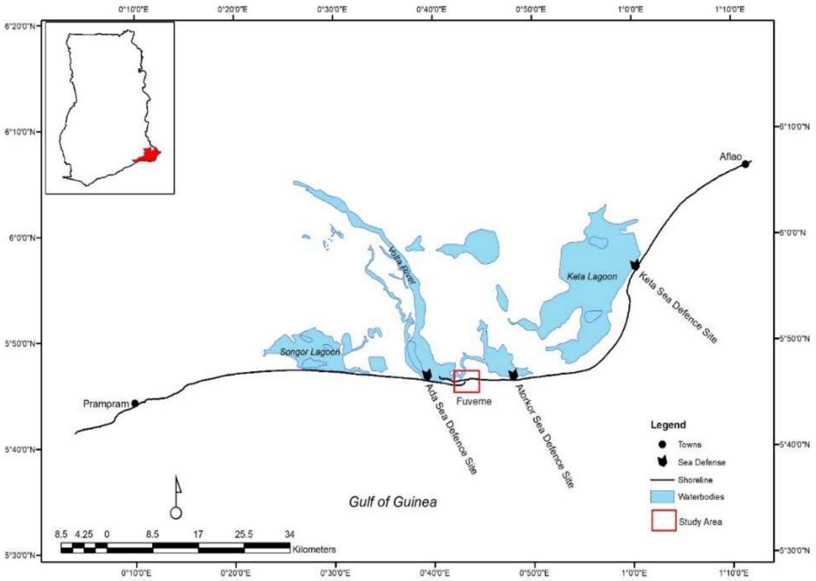

Figure 1: Location of the Study Area

Table 1: Satellite Imagery and Orthophoto Information

Imagery Type	Fuvemeh
World View -2<br>(0.46m Pan & 1.84m Multispectral)	15/07/2017<br>30/09/2014<br>28/02/2014<br>10/09/2013<br>20/03/2013<br>23/12/2012<br>04/07/2010
WorldView -3<br>(0.31m Pan & 1.24m Multispectral)	16/03/2016<br>02/11/2014
GeoEye -1<br>(0.41m Pan & 1.65m Multispectral)	17/10/2009
Orthophoto (0.50m RGB)	2005

*Dates in dd/mm/yyyy

3.0 Shoreline and building extraction

The extracted shorelines were used to statistically (linear regression) estimate the historic rate of shoreline change by casting perpendicular transects at 20 m intervals alongshore. The AMBUR functions (Jackson et al., 2012) was adopted for the rates of change computation. Shoreline positional accuracy was determined by accounting for four main sources of error. They include the ground sampling distance (E_p), geo-referencing error (average RMSE from geo-referencing-E_g), the digitizing error (E_d) estimated to be 1.5 of a pixel, and tidal error (E_t) (Ford, 2013; Li et al., 2001). The total positional error ($\mathrm{E_r}$) for each shoreline was estimated using the expression:

$$E_r = \sqrt{(E_p^2 + E_g^2 + E_d^2 + E_t^2)}$$

An annualized error was estimated to account for the uncertainty in the rate of change at each transect.

4.0 Results and Discussion

The shoreline exhibited a highly dynamic characteristic (Figure 2). Overall, erosion and accretion rates averaged at 7.24m/year and 5.83m/year respectively with the net erosion rate of 1.41 m/year, which is relatively high. It emerged that about 52% of the shoreline is experiencing deposition while the remainng 48% is eroding at varying intensity. Analysis of the shoreline rates

of change between 2005 and 2013, which is the period before the construction of the Ada sea defense structures, revealed that the shoreline in Fuvemeh experienced accretion (56%) at a rate of about 4.4m/year. However, the period after the construction of the Ada sea defense structures (between 2013 and 2017) experienced erosion at an average rate of about 31m/year. Increased flooding has been observed in the community and the frequency has increased in recent years (Appeaning Addo et al., 2018). Additionally, the Analysis of the results show that the shoreline has moved inland by over 100 along some transects. It also emerged that about 77 houses have been destroyed suggesting the displacement of over 300 inhabitants. Analysis of changes in features identified on the satellite imageries revealed that the position of the mouth of the Volta river is influenced by a dynamic sandbar on the eastern side of the estuary, which breaches and form new features.

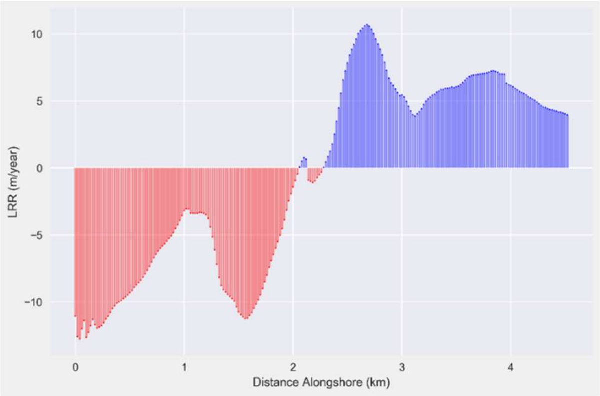

Figure 2: Erosion trends along the Fuvemeh Coastline showing the overall changes (2005 to 2017)

5.0 Conclusion

The study has revealed erosion trend and flooding impact on the vulnerable Fuvemeh coastal community in the Volta delta. It is projected that more infrastructures will be destroyed, which will result in increased migration from the Fuvemeh community. Destruction of livelihoods will increase unemployment and increase poverty. There is therefore the need to relocate the Fuvemeh community to a safer location. Effective adaptation approaches to increase the resilience of the inhabitants in the community should also be developed.

6.0 References

Mumuni Abu, Yaw Atiglo, Cynthia Addoquaye-Tagoe, Samuel Codjoe (2017). Descriptive Statistics from Sending Area Survey Data - Volta Delta, Ghana. DECCMA consortium meeting, Bngladesh. http://www.deccma.com/deccma/Working_Papers/ 19/10/2018.

Angnuureng, D. B., Appeaning Addo, K., & Wiafe, G. (2013). Impact of sea defense structures on

downdrift coasts: The case of Keta in Ghana. *Academia Journal of Environmental Sciences*, *1*(6), 104–121. http://doi.org/http://dx.doi.org/10.15413/ajes.2013.0102

Appeaning Addo, K., Walkden, M., & Mills, J. P. (2008). Detection, measurement and prediction of shoreline recession in Accra, Ghana. *ISPRS Journal of Photogrammetry and Remote Sensing*, *63*(5), 543–558. http://doi.org/10.1016/j.isprsjprs.2008.04.001

Boateng, I. (2012a). An application of GIS and coastal geomorphology for large scale assessment of coastal erosion and management: A case study of Ghana. *Journal of Coastal Conservation*, *16*(3), 383–397. http://doi.org/10.1007/s11852-012-0209-0

Boateng, I., Bray, M., & Hooke, J. (2012b). Estimating the fluvial sediment input to the coastal sediment budget: A case study of Ghana. *Geomorphology*, *138*(1), 100–110. http://doi.org/10.1016/j.geomorph.2011.08.028

Bollen M, Trouw K, Lerouge F, Gruwez V, Bolle A, Hoffman B, Mercelis P (2011) Design of a Coastal Protection Scheme for Ada at the Volta-River Mouth (Ghana). Coastal Engineering Proceedings, 1(32): 36.

Ford, M. (2013). Remote Sensing of Environment Shoreline Changes Interpreted from Multi-Temporal Aerial Photographs and High Resolution Satellite Images?: Wotje Atoll, *135*, 130–140.

Ghana Statistical Service. (2012). *2010 Population and Housing Census*. Accra.

Jackson, C. W., Alexander, C. R., & Bush, D. M. (2012). Application of the AMBUR R package for spatio-temporal analysis of shoreline change: Jekyll Island, Georgia, USA. *Computers and Geosciences*, *41*, 199–207. http://doi.org/10.1016/j.cageo.2011.08.009

Jayson-Quashigah, P.-N., Appeaning Addo, K., & Kufogbe, S. K. (2013). Medium resolution satellite imagery as a tool for monitoring shoreline change. Case study of the Eastern coast of Ghana. *Journal of Coastal Research*, *65*(SI), 511–516. http://doi.org/10.2112/SI65-087.1

Li, R., Di, K., & Ma, R. (2001). A Comparative Study of Shoreline Mapping Techniques. *Geology*. http://doi.org/doi:10.1201/9781420023428.ch3\r10.1201/9781420023428.ch3

Ly, C. K. (1980). The role of the Akosombo Dam on the Volta River in causing coastal erosion in central and eastern Ghana (West Africa). *Marine Geology*, *37*, 323–332.

Wiafe, G., Boateng, I., Appeaning-Addo, K., Jayson-Quashigah, P. N., Ababio, S. D., & Sowah, L. (2013). *Handbook Of Coastal Proccesses And Management In Ghana* (1st ed.). The Choir Press.

Fuvemeh, Ghana

TABLE 1: Typology to assess the hazards and susceptibility of a coastal locality

#	Hazard and Susceptibility Elements	Indicators / Metrics	Sources
1	**Settlement location**	0.6898° to 0.7107° E 5.7706° to 5.7754° N, 0km from shoreline with others less than 0.1km, relaitve sea level rise (ref to 2000): +0.21-0.36m for mid-century and +0.55-1.1m by end-century	- Kebede, A. S., Nicholls, R. J., Allan, A., Arto, I., Cazcarro, I., Fernandes, J. A., … Whitehead, P. W. (2018). Applying the global RCP – SSP – SPA scenario framework at sub-national scale : A multi-scale and participatory scenario approach. Science of the Total Environment, 635(March), 659–672.
2	**Köppen–Geiger climate classification system**	Present: Bsh-Arid, Steppe, Hot arid; A1FI Future Scenarios (2001-2050): Aw-Equatorial winter dry; (2051-2100) As-Equatorial steppe	- Kottek, M., J. Grieser, C. Beck, B. Rudolf, and F. Rubel, 2006: World Map of the Köppen-Geiger climate classification updated. Meteorol. Z., 15, 259-263. DOI: 10.1127/0941-2948/2006/0130.
3	**Isostatic rebound**	Not Applicable	
4	**Subsidence**	Not measured – probably 1 to 2 mm/yr based on other deltas. It may accelerate due to human effects.	- Syvitski, J.P.M, 2008. Deltas at risk. Sustainability Science, 3(1), 23-32.https://doi.org/10.1007/s11625-008-0043-3.
5	**Local/regional mass density changes**	SLR Presently 3.1 mm/yr and expected to accelerate significantly.	- Church el al., 2013;<br>- Sagoe-Addy and Appeaning Addo, 2013
6	**Coastal erosion**	7.24m/year and no mitigation managament on site	- DECCMA Project, 2018
7	**Slopes and angles on or near the shore**	Average beach less than 100m with upper beach slope 1:3 and lower beach 1:25	- DECCMA Project, 2018<br>- Roest, L. W. M. (2018). The coastal system of the Volta delta , Ghana Opportunities and strategies for development.
8	**Located in tropical or other storm zone**	Not Applicable	
9	**Inland Rainfall**	Ada Station closest. IDF graphs for Ada	- Logah, F. Y., Kankam-Yeboah, K., & Bekoe, E. O. (2013). Developing short duration rainfall intensity frequency curves for Accra in Ghana. International Journal of Research in Engineering and Computing, 1(1), 67-73.
10	**Inland rivers**	Aksombo dam built in 1964 on the Volta river. Major impacts on freshwater inputs and especially sediment supply, which has been greatly reduced.	- Woodroffe, C.D., 2010. Assessing the vulnerability of Asian megadeltas to climate change using GIS. In: Green, D.R. (ed.), Coastal and Marine Geospatial Technologies. Dordrecht, Netherlands: Springer, pp. 379–391.
11	**Extent and likelihood of coastal and/or fluvial flooding**	Entire community experience flooding due to tidal waves	- Kwasi Appeaning Addo, Philip-Neri Jayson-Quashigah, Samuel Nii Ardey Codjoe, Francisca Martey (2018). Drone as a tool for Coastal Flood Monitoring in the Volta delta, Ghana. Geoenvironmental Disasters. 5:17 DOI 10.1186/s4077-018-0108-2
12	**Air temperature**	Not Documented	
13	**Ocean/Coastal Parameters**	Guinea Current (West to East) 0.5-1.5m/s; Sea Temperature 23-28 Degrees Celcius; Salinity is estimated to be between 34.90-35.60 per mille	- Giardino, A., Schrijvershof, R., Nederhoff, C. M., de Vroeg, H., Brière, C., Tonnon, P. K., ... & Schellekens, J. (2018). A quantitative assessment of human interventions and climate change on the West African sediment budget. Ocean & Coastal Management, 156, 249-265.<br>- https://seatemperature.info/ghana-water-temperature.html and<br>- Odekunle, T. O., & Eludoyin, A. O. (2008). Sea surface temperature patterns in the Gulf of Guinea: their implications for the spatio-temporal variability of precipitation in West Africa. International Journal of Climatology, 28(11), 1507-1517.<br>- Dovlo (2016) seasonal variations in temperature and salinity in the Gulf of Guinea. Journal of Aquaculture and Marine Biology, 4(2)
14	**Habitats**	100,000 square meters of mangrove area, 200, 000 square meters of sand dune	- Estimated from Google earth
15	**Groundwater salinization**	Ground water production is less than 1% of groundwater recharge in the Basin. Mean groundwater storage 1000km^3	- Williams et al,. 2016. The volta River Basin<br>- Nicola Martin & Nick van de Giesen (2005) Spatial Distribution of Groundwater Production and Development Potential in the Volta River basin of Ghana and Burkina Faso, Water International, 30:2, 239-249, DOI: 10.1080/02508060508691852
16	**Base Rock**	Quartz-biotite gneiss (Dahomeyan) beyond 4km depth	- Akpati (1978) Geologic Structure and evolution of the Keta basin Ghana, West Africa
17	**Other non-coastal natural hazards**	Not Applicable	

Fuvemeh, Ghana

Table 2: Typology to assess exposure and vulnerability of a coastal locality

#	Exposure and Vulnerability Elements	Indicators / Metrics	Sources
18	**Population**	836 current estimates	- Ghana Population and Housing Census 2010
19	**Future Population Change**	Estimated to decrease as a result of coastal erosion and flooding	- Kwasi Appeaning Addo, Philip-Neri Jayson-Quashigah, Samuel Nii Ardey Codjoe, Francisca Martey (2018). Drone as a tool for Coastal Flood Monitoring in the Volta delta, Ghana. Geoenvironmental Disasters. 5:17 DOI 10.1186/s4077-018-0108-2
20	**Historic coastal and/or fluvial flooding**	Whole community experiences coastal flooding frequently. Over 77 houses have been destroyed between 2005 and 2017, which has resulted in the displacement of over 300 inhabitants	- Kwasi Appeaning Addo, Philip-Neri Jayson-Quashigah, Samuel Nii Ardey Codjoe, Francisca Martey (2018). Drone as a tool for Coastal Flood Monitoring in the Volta delta, Ghana. Geoenvironmental Disasters. 5:17 DOI 10.1186/s4077-018-0108-2
21	**Human Development Index (national)**	0.592	UNDP (2018) Ghana [WWW Document]. URL: http://hdr.undp.org/sites/all/themes/hdr_theme/country-notes/GHA.pdf
22	**GNP/capita (probably national)**	$2,046 (2017)	World Bank (2018) World Bank Open Data. URL: https://data.worldbank.org/
23	**Proportion of national population that is coastal**	A quarter of population is coastal	- Appeaning Addo, K., Walkden, M., & Mills, J. P. (2008). Detection, Measurement and Prediction of Shoreline Recession in Accra, Ghana. ISPRS Journal of Photogrammetry and Remote Sensing. 63(5): 543-558.
24	**Governance**	Traditional Chief and Assemble Member	- Local knowledge
25	**Relationships to larger governmental entities**	Keta Municipal Assembly	- Local knowledge
26	**Relationships to international entities**	National Disaster Management Organisation (NADMO)	- Local knowledge
27	**Built Infrastructure**	About 200 buildings sparsely distributed in the community; No motorable road, basic school	- Local knowledge
28	**Natural Capital**	Destruction of coconut trees and mangrove; reduced marine fish stock; loss of income due to increased erosion and flooding	- Appeaning Addo, K., Nicholls, R. J., Nii, S., Codjoe, A., & Abu, M. (2018). A Biophysical and Socioeconomic Review of the Volta Delta , Ghana. Journal of Coastal Research, In-Press. http://doi.org/10.2112/JCOASTRES-D-17-00129.1
29	**Available geographic/GIS data**	Orthophoto and DEM	- Ghana Survey<br>- DECCMA Project
30	**Minority status**	The indigenous people are Ewe; major religion is christianity;	- Ghana Population and Housing Census 2010
31	**Historical areas**	Not Applicable	
32	**Environmental areas**	Turtle Nesting site (not protected)	- Local knowledge
33	**Cultural areas**	Fishing community	- Local knowledge
34	**Tourism areas**	Close to the Volta Estuary	- Local knowledge
