# Supplementary material for: Challenges to climate change adaptation in coastal small towns: Examples from Ghana, Uruguay, Finland, Denmark, and Alaska
Source: Ocean Coast Manag. 2021 Oct 15;212:105787. doi: 10.1016/j.ocecoaman.2021.105787 (PMC10644629; doi:10.1016/j.ocecoaman.2021.105787)
Supplement: Multimedia component 2 [file mmc2.pdf]

A typology of small coastal towns and cities for climate adaptation planning: Finnish case

Samrit Luoma[1*] and Johannes Klein[1]

[1]Geological Survey of Finland, P.O. Box 96, 02151 Espoo, Finland

Abstract

The town of Hanko is located in a low-lying coastal area and has population of less than 10,000. It is a popular summer holiday place for holiday homeowners and tourists. The town lies above an important groundwater area that is vulnerable to climate change and anthropogenic impacts. This study provides a typology to assess Hanko's exposure and vulnerability to climate change and its challenges and opportunities for adaptation. The typology contains a clear list of key components needed for climate impact assessment and adaptation in the Hanko area.

1.0 Introduction

The town of Hanko is located on the Hanko Cape at the southern coast of Finland (approx. 59º53"N 23º10"E, Figure 1). It covers 117 km^2 of land area and 683 km^2 of the Baltic Sea area. The population of Hanko was 8379 at the end of 2018, and according to projections, it will decrease to6119 by 2040 (Hanko 2020, Statistics Finland 2020). The economy of Hanko is based on services (61%) and industry (38%). The bilingual port town of Hanko (Finnish and Swedish speaking) (founded in 1874) has a long history dated back at least to the 13th century. Together with the local culture, the unique nature and the long sandy beaches make Hanko a popular summer resort, where the population increases considerably during the summer due to the arrival of holiday homeowners and tourists.

The Hanko Cape belongs to the temperate coniferous-mixed forest climate zone with cold and wet winters. According to Köppen-Geiger climate classification, this climate is classified as Dfb (Kottek et al. 2006). The mean annual temperature is 6 ºC, with mean minimum and maximum temperatures of -4.2 and 16.6 ºC, respectively. The average annual precipitation was 634 mm during the period 1981–2010 (Pirinen et al. 2012). The area is relatively flat with the average elevation of 12-14 m a.s.l. The Hanko Cape contains an important groundwater aquifer which consists of porous gravels and sands of an ice-marginal end-deposit and is bounded by the Baltic Sea (Luoma et al.

*Corresponding Author: samrit.luoma@gtk.fi

2013; 2014). It is an important source of water for the residents of the Hanko and the local industries. However, in some parts the groundwater table is close to the ground surface. A rise in sea level would cause some parts of the Hanko aquifer to be below sea level which may affect groundwater quantity and quality due to salt water intrusion. This, together with the predicted increase in precipitation, would increase groundwater recharge and raise the water table, consequently contributing to the potential deterioration of groundwater quality or potential flooding in the low-lying aquifer area. The Hanko Cape is part of EU and Finland's Natura 2000 network, which hosts a number of important species and habitats, e.g. many species of fish, birds. Climate change and human activities in the future may threaten those species and ecosystems in the coastal areas.

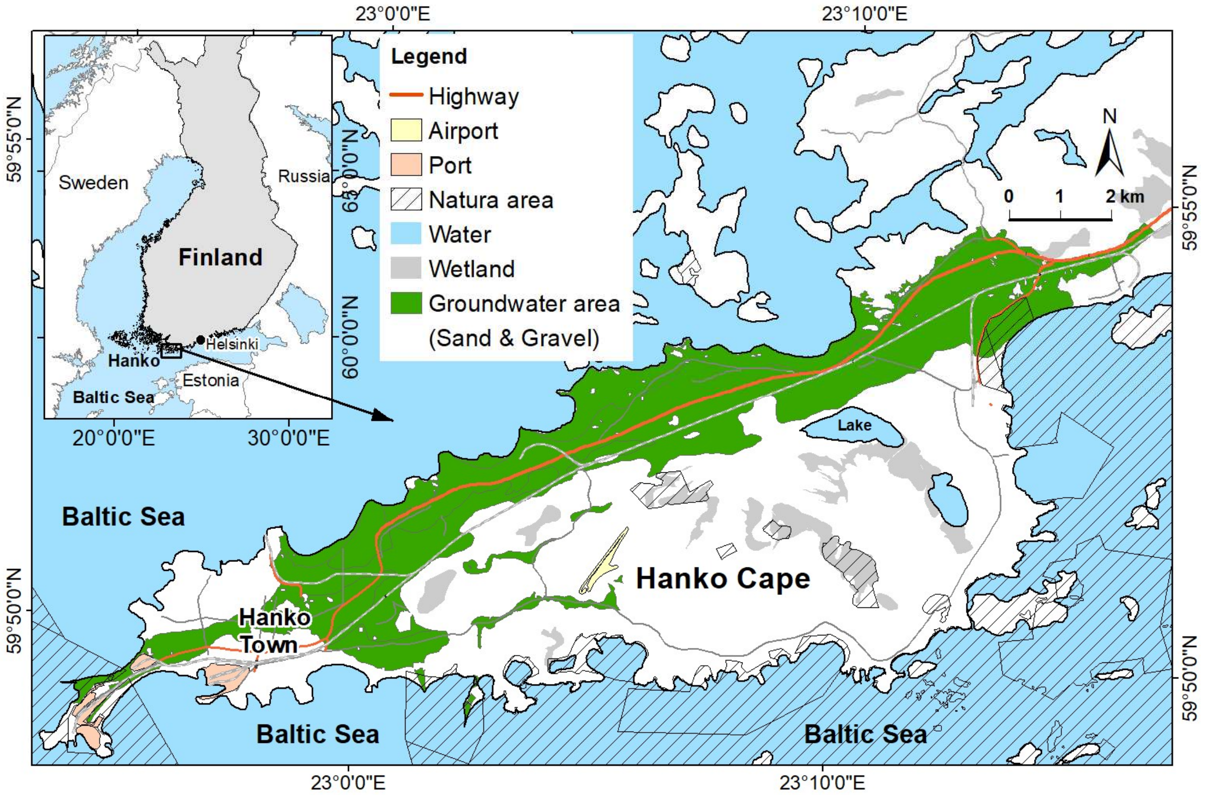

Figure 1: Location map of the study area in Hanko, south Finland. Basemap @ National Land Survey of Finland and HALTIK.

The Hanko Cape belongs to the temperate coniferous-mixed forest climate zone with cold and wet winters. According to Köppen-Geiger climate classification, this climate is classified as Dfb (Kottek et al. 2006). The mean annual temperature is 6 ºC, with mean minimum and maximum temperatures of -4.2 and 16.6 ºC, respectively. The average annual precipitation was 634 mm during the period 1981–2010 (Pirinen et al. 2012). The area is relatively flat with the average elevation of 12-14 m a.s.l. The Hanko

Cape contains an important groundwater aquifer which consists of porous gravels and sands of an ice-marginal end-deposit and is bounded by the Baltic Sea (Luoma et al. 2013; 2014). It is an important source of water for the residents of the Hanko and the local industries. However, in some parts the groundwater table is close to the ground surface. A rise in sea level would cause some parts of the Hanko aquifer to be below sea level which may affect groundwater quantity and quality due to salt water intrusion. This, together with the predicted increase in precipitation, would increase groundwater recharge and raise the water table, consequently contributing to the potential deterioration of groundwater quality or potential flooding in the low-lying aquifer area. The Hanko Cape is part of EU and Finland's Natura 2000 network, which hosts a number of important species and habitats, e.g. many species of fish, birds. Climate change and human activities in the future may threaten those species and ecosystems in the coastal areas.

2.0 Typology of Hanko

The typology of the Hanko town for climate adaptation planning is summarized in Table 1 and Table 2. Additional information concerning the studies of climate change adaptation in Hanko are presented in the following sections:

2.1. Availability and sources of climate scenarios

Based on the Fifth Assessment Report of the Intergovernmental Panel on Climate Change (IPCC), two climate change projections are available in Finland, the RCP4.5 scenario representing fairly moderate emissions and the RCP8.5 scenario representing high emissions. These two climate scenarios have the mean summer temperature projections as much as 1°C higher than the previous model ensemble (which was used to prepare the IPCC's 4th Assessment Report - SRES) (Ministry of the Environment and Statistics Finland 2017). Luoma et al. (2013) assessed potential climate change for Hanko based on the SRES A1B and B1 scenarios which were available during that time. These two climate model data A1B and B1 scenarios for the period 2001–2100 and a simulation of present climate 1971–2000 were obtained from the regional climate model CLM (Climate Limited-area Modelling community of the German Meteorological Service) from the World Data Center for Climate in Hamburg (Hollweg et al. 2008). The boundary conditions of the CLM model were derived from the ECHAM5/MPI-OM global climate model of the Max Planck Institute

for Meteorology. The sea-level rise scenarios were also available for the A1B and B1 from the CLIMBER model, with compensation for the vertical post-glacial land movement component for the period 2000–2100 with the baseline data of 1995 (Petoukhov et al. 2000, Ganopolski et al. 2001). Daily weather data measured at the Tvärminne weather station in Hanko since 1963, are available from the Finnish Meteorological Institute (FMI). The longest time series of sea level data along the coast of Finland has been measured since 1887 at the tide gauge in Hanko, and daily sea-level data are available from the FMI - Marine Research.

2.2. Climate projections for Hanko

The greatest increase in mean temperature in Hanko is expected to be 2.6-4.6 ºC, with a mean annual increase of 3.4 ºC by the end of the 21st century under the A1B scenario. The maximum increase in mean temperature (3.7-4.6 ºC) is expected to take place in winter and the minimum (2.6-2.9 ºC) in summer. The annual precipitation increased by 5-12% with the highest increase (2-26%) in autumn and winter, while in summer, mean precipitation decreased by 0-10% compared with 1971-2000 (Luoma et al. 2013, Luoma & Okkonen 2014). As a consequence, it is expected that heat waves and heavy precipitation events will intensify. The number of days with precipitation will increase in winter, while the snow season will become shorter. Post-glacial land uplift compensates some of the expected future sea level rise in this area. However, the predicted increases in the number of the extreme events such as the storm surges induced sea level rise and coastal inundation cause the Hanko town more vulnerable. Based on the study of Luoma and Okkonen (2014), the mean sea level in the Hanko area is predicted to reach +0.51 m a.s.l. under the sea-level rise scenario A1B and the potential storm surges would reach 1.75 m a.s.l. by the end of the 21st century. At this level, the areas below +0.51 m a.s.l., e.g. low-lying area along the coast and ports would be under water, and the areas below 1.75 m a.s.l., including the city's water intake wells will be vulnerable to coastal flooding. This will have the impacts not only on the land use planning, but also the drinking water resources. Because the aquifer in Hanko is located in a cold snow-dominated region, the seasonal impacts of climate change on groundwater recharge were more significant, with land surface overflow resulting in flooding during the winter and early spring and drought during the summer. Rising sea levels would cause some parts of the aquifer

to be submerged under the sea, compromising groundwater quality due to the intrusion of seawater.

2.3. Vulnerability, risks and climate change impacts

Many studies have been carried out for the assessment of climate change impacts, risks, vulnerabilities and adaptations in Finland (e.g. Ministry of the Environment and Statistics Finland 2017, FINADAPT 2007). The overall vulnerability to coastal flooding and erosion due to sea level rise is reported to be low. However, in the Hanko area with the unique feature of the relatively low topography and the long sandy beaches, rising sea levels and storm surges will increase more the flood-risk and erosion along the coast than in other parts of Finland, which may also impact freshwater availability or loss of coastal eco-systems in flood-prone areas. By the end of the 21st century, the density and salinity of the Baltic Sea water are predicted to be the same as present or lower due to the increasing input of freshwater into the Baltic Sea. The degree of seawater intrusion is therefore probably not the main concern compared with coastal flooding of the aquifer due to sea-level rise and storm surges. In the Hanko area, however, overpumping due to the seasonal increase in the groundwater demand during the summer with the increased number of tourists and/or the owners of summer cottages, could lower the groundwater level, and may cause the aquifer to become more vulnerable to seawater intrusion. Under drought conditions, this could lead to a potential water shortage during the summer (Luoma et al. 2013; 2015; 2017, Luoma & Okkonen 2014).

2.4. Funding sources for mitigation and adaptation activities

In Finland, coastal zone management is a responsible of municipalities, while the national authorities provide guidelines with respect to land use planning and Regional Environment Centres recommend about minimum construction heights in coastal and other flood-prone areas. The municipalities will decide whether or not they include safety margins into their land use plans and obtain later on permits at the national level. Therefore, the costs of coastal protection measures and management are the responsible of municipalities (European Commission 2009). The city of Hanko has participated in many climate adaptation projects at national and international level. For example, the BaltCICA project (2009-2012) has raised the awareness of the climate change impacts on the spatial planning and water resources to the municipalities,

stake-holder and local people (Luoma et al. 2013). Many projects have later on been planned and implemented such as the testing area for artificial groundwater, the cooperation with the neighbor town on the water supply and management issues, detailed investigations of hydrogeology of the aquifers including the update of vulnerability and risk assessments of groundwater areas for better groundwater protection plans and managements to ensure that there is enough clean water to the communities in the future.

2.5. Relations to higher-level governmental/international/NGO groups and agencies

According to the European Commission report (European Commission (2009), there is no national plan for coastal protection in Finland. The planning and implementation of coastal protection measures is the responsibility of the municipalities. In Hanko, the Uusimaa Centre for Economic Development, Transport and the Environment (ELY Centres - Uusimaa) and the city of Hanko develop their spatial plans and implement coastal adaptation measures in line with the National Land Use and Building Act, Guidelines and the National Adaptation Strategy. Hanko takes responsibility for cost and planning for the coastal management in the Hanko area. The ELY Centres - Uusimaa is responsible for steering land use planning both in the regional and national guidelines, and also involved in environmental protection, nature protection and water resources management. The Finnish Environment Institute (SYKE) is a key operator in protecting species and habitats in the natural areas. Moreover, many EU projects and the private agencies have been actively operated in the area in order to keep the Baltic Sea clean and safe at present and future climate conditions.

3.0 Conclusion

The typology was developed for small coastal towns such as Hanko for the assessment of exposure and vulnerability to climate change and its challenges and opportunities for adaptation. In Hanko, groundwater is an important source for public water supply and local industries. However, it is vulnerable to climate change and anthropogenic risks. Post-glacial land uplift compensates some of the expected future sea level rise. However, the predicted increases in the number of the extreme events such as the storm surges and coastal inundation cause Hanko to be vulnerable to the impacts of climate change. Hanko faces typical challenges for coastal small towns, such as restricted resources and access to suitable local climate data. However, joint

projects with research organisations and cooperation with other towns provide also adaptation opportunities.

4.0 References

European Commission. 2009. The economics of climate change adaptation in EU coastal areas – Finland Country report. The European Commission. [WWW Document]. URL: https://ec.europa.eu/maritimeaffairs/documentation/studies/climate_change_en

Eurostat. 2020. The European statistical data. [WWW Document]. URL: https://ec.europa.eu/eurostat

FINADAPT. 2007. Assessing the adaptive capacity of the Finnish environment and society under a changing climate (FINADAPT). Finnish Environment Institute (SYKE) [WWW Document]. URL: http://www.syke.fi/projects/finadapt

Finnish Government. 2020. Information of the Finnish Government. [WWW Document]. URL: https://valtioneuvosto.fi/en/government

FMI. 2020a. Finnish Meteorological Institute (FMI) - Marine research. [WWW Document]. URL: https://en.ilmatieteenlaitos.fi/marine-research

GTK. 2020. Bedrock map of Finland at scale 1: 1 000 000. Geological Survey of Finland. Espoo. Finland.

Hanko. 2020. Information of the Hanko City. [WWW Document]. URL: www.hanko.fi

Hollweg, H. D., Böhm, U. Fast, I., Hennemuth, B., Keuler, K., Keup-Thiel, E., Lautenschlager, M., Legutke, S., Radtke, K., Rockel, B., Schubert, M., Will, A., Woldt, M. & Wunram, C. 2008. Ensemble Simulations over Europe with the Regional Climate Model CLM forced with IPCC AR4 Global Scenarios. The Model and Data Technical Report No.3. Max Planck Institute for Meteorology, Hamburg. [WWW Document]. URL: https://www.dkrz.de

Kottek, M., Grieser, J., Beck, C., Rudolf, B. & Rubel, F. 2006. World Map of the Köppen-Geiger climate classification updated. *Meteorol. Z.*, 15, 259-263. doi: 10.1127/0941-2948/2006/0130.

Luke. 2020. Natural resources of Finland. [WWW Document]. URL: https://www.luke.fi/en/natural-resources/

Luoma, S., Klein, J. & Backman, B. 2013. Climate change and groundwater: Impacts and Adaptation in shallow coastal aquifer in Hanko, south Finland. *In*: Schmidt-Thomé, P. & Klein, J. (eds) Climate Change Adaptation in Practice – From Strategy Development to Implementation. Wiley-Blackwell, 137−155.

Luoma, S. & Okkonen, J. 2014. Impacts of Future Climate Change and Baltic Sea Level Rise on Groundwater Recharge, Groundwater Levels, and Surface Leakage in the Hanko Aquifer in Southern Finland. Water 6:3671-3700, doi:10.3390/w6123671

Luoma, S., Okkonen, J., Korkka-Niemi, K., Hendriksson, N. & Backman, B. 2015. Confronting vicinity of the surface water and sea shore in a shallow glaciogenic aquifer in southern Finland, Hydrol. Earth Syst. Sci. 19:1353-1370, doi:10.5194/hess-19-1353-2015

Luoma, S., Okkonen, J. & Korkka-Niemi, K. 2017. Comparison of the AVI, modified SINTACS and GALDIT vulnerability methods under future climate-change scenarios for a shallow low-lying coastal aquifer in southern Finland. Hydrogeology J. 25: 203. doi.org/10.1007/s10040-016-1471-2

Ministry of the Environment. 2020. The protected nature areas in Finland. [WWW Document]. URL: http://www.environment.fi/en- US/Nature/Protected_areas

Ministry of the Environment and Statistics Finland. 2017. Finland's Seventh National Communication under the United Nations Framework Convention on Climate Change. Ministry of the Environment and Statistics Finland. 314 p. [WWW Document]. URL:

https://www.stat.fi/static/media/uploads/tup/khkinv/VII_Climate_Change_16102017.pdf

NLS. 2020. National Land Survey of Finland (NLS). [WWW Document]. URL: https://www.maanmittauslaitos.fi/en

Pirinen, P., Simola, H., Aalto, J., Kaukoranta, J-P., Karlsson, P. & Ruuhela, R. 2010. Climatological statistics of Finland 1981–2010. Finnish Meteorological Institute. [WWW Document]. URL: https://helda.helsinki.fi/handle/10138/35880

Statistics Finland. 2020a. Population and Population projection of Finland. [WWW Document]. URL: https://www.stat.fi/til/vrm_en.html

Statistics Finland. 2020b. Annual national accounts. [WWW Document]. URL: http://www.stat.fi/til/vtp/index_en.html

UNDP. 2020. Human Development Report: Finland. The United Nations Development Programme. [WWW Document]. URL: http://hdr.undp.org/en/countries/profiles/FIN

World Bank. 2020. The World Bank Data. [WWW Document]. URL: http://data.worldbank.org/indicator/NY.GNP.PCAP.PP.CD?view=map

Hanko, Finland

Table 1: Typology to assess the hazards and susceptibility of a coastal locality

#	Hazard and Susceptibility Elements	Indicators / Metrics	Sources
1	**Settlement location**	The city of Hanko, south Finland at approx. 59º53”N 23º10”E; size of land 117 km^2, surrounded by the Baltic Sea. Elevations vary from zero to 14 m a.s.l., the projected mean sea level rise at +0.51 m by the end of 2100.	- NLS. 2020. National Land Survey of Finland (NLS). [WWW Document]. URL: https://www.maanmittauslaitos.fi/en<br>- Hanko. 2020. Information of the Hanko City. [WWW Document]. URL: www.hanko.fi<br>- Hollweg, H. D., Böhm, U. Fast, I., Hennemuth, B., Keuler, K., Keup-Thiel, E., Lautenschlager, M., Legutke, S., Radtke, K., Rockel, B., Schubert, M., Will, A., Woldt, M. & Wunram, C. 2008. Ensemble Simulations over Europe with the Regional Climate Model CLM forced with IPCC AR4 Global Scenarios. The Model and Data Technical Report No.3. Max Planck Institute for Meteorology, Hamburg. [WWW Document]. URL: https://www.dkrz.de
2	**Köppen–Geiger climate classification system**	Dfb-Warm humid continental climate; remains the same in the future.	- Kottek, M., Grieser, J., Beck, C., Rudolf, B. & Rubel, F. 2006. World Map of the Köppen-Geiger climate classification updated. Meteorol. Z., 15, 259-263. doi: 10.1127/0941-2948/2006/0130.
3	**Isostatic rebound**	Land uplift in the Hanko Cape is approx. 0.43 cm/yr relative to the centre of the Earth.	- NLS. 2020. National Land Survey of Finland (NLS). [WWW Document]. URL: [illegible]
4	**Subsidence**	No subsidence in the area.	- NLS. 2020. National Land Survey of Finland (NLS). [WWW Document]. URL: https://www.maanmittauslaitos.fi/en
5	**Local/regional mass density changes**	The sea level rise is about 0.15 cm/yr. Increases in number of storm surges induced sea level rise and coastal inundation.	- NLS. 2020. National Land Survey of Finland (NLS). [WWW Document]. URL: https://www.maanmittauslaitos.fi/en
6	**Coastal erosion**	Coastal erosion in the sand/gravel beaches by winds and waves.	- NLS. 2020. National Land Survey of Finland (NLS). [WWW Document]. URL: https://www.maanmittauslaitos.fi/en
7	**Slopes and angles on or near the shore**	Sand beaches 30 km long, spatially variations of beaches and near slopes from less than 1° to over 10°.	- NLS. 2020. National Land Survey of Finland (NLS). [WWW Document]. URL: https://www.maanmittauslaitos.fi/en
8	**Located in tropical or other storm zone**	The average sea water temperatures are below 26ºC.	- FMI. 2020a. Finnish Meteorological Institute (FMI) - Marine research. [WWW Document]. URL: https://en.ilmatieteenlaitos.fi/marine-research
9	**Inland Rainfall**	Mean precipitations increase 10-25% by the end of 2100 compared with present (1971-2000). Potential seasonal flooding during spring and drought during summer.	- FMI. 2020b. Finnish Meteorological Institute (FMI) [WWW Document]. URL: www.fmi.fi<br>- Hollweg, H. D., Böhm, U. Fast, I., Hennemuth, B., Keuler, K., Keup-Thiel, E., Lautenschlager, M., Legutke, S., Radtke, K., Rockel, B., Schubert, M., Will, A., Woldt, M. & Wunram, C. 2008. Ensemble Simulations over Europe with the Regional Climate Model CLM forced with IPCC AR4 Global Scenarios. The Model and Data Technical Report No.3. Max Planck Institute for Meteorology, Hamburg. [WWW Document]. URL: https://www.dkrz.de<br>- Luoma, S., Klein, J. & Backman, B. 2013. Climate change and groundwater: Impacts and Adaptation in shallow coastal aquifer in Hanko, south Finland. In: Schmidt-Thomé, P. & Klein, J. (eds) Climate Change Adaptation in Practice – From Strategy Development to Implementation. Wiley-Blackwell, 137−155.<br>- Luoma, S. & Okkonen, J. 2014. Impacts of Future Climate Change and Baltic Sea Level Rise on Groundwater Recharge, Groundwater Levels, and Surface Leakage in the Hanko Aquifer in Southern Finland. Water 6:3671-3700, doi:10.3390/w6123671
10	**Inland rivers**	No rivers in the area, but a small lake (1.18 km^2) with wetlands in the low-lying areas. Low-lying areas have been ditched out to the sea to prevent the flooding.	- Hanko. 2020. Information of the Hanko City. [WWW Document]. URL: www.hanko.fi<br>- Luoma, S., Klein, J. & Backman, B. 2013. Climate change and groundwater: Impacts and Adaptation in shallow coastal aquifer in Hanko, south Finland. In: Schmidt-Thomé, P. & Klein, J. (eds) Climate Change Adaptation in Practice – From Strategy Development to Implementation. Wiley-Blackwell, 137−155.<br>- Luoma, S. & Okkonen, J. 2014. Impacts of Future Climate Change and Baltic Sea Level Rise on Groundwater Recharge, Groundwater Levels, and Surface Leakage in the Hanko Aquifer in Southern Finland. Water 6:3671-3700, doi:10.3390/w6123671<br>- Luoma, S., Okkonen, J., Korkka-Niemi, K., Hendriksson, N. & Backman, B. 2015. Confronting vicinity of the surface water and sea shore in a shallow glaciogenic aquifer in southern Finland, Hydrol. Earth Syst. Sci. 19:1353-1370, doi:10.5194/hess-19-1353-2015<br>- Luoma, S., Okkonen, J. & Korkka-Niemi, K. 2017. Comparison of the AVI, modified SINTACS and GALDIT vulnerability methods under future climate-change scenarios for a shallow low-lying coastal aquifer in southern Finland. Hydrogeology J. 25: 203. doi.org/10.1007/s10040-016-1471-2

11	**Extent and likelihood of coastal and/or fluvial flooding**	The highest storm surges occured 1.21 times during the past 100 years, which regarded as a low impact. In the future storm surges and flood event are expected to be intensify due to the projected increase in precipitations.	- FMI. 2020b. Finnish Meteorological Institute (FMI) [WWW Document]. URL: www.fmi.fi
12	**Air temperature**	At present heat waves have increased, but no statistically significant trend, due to the large variability of the weather in the region. The projected climate change indicates increasing frequency and length of heat aves, which could be threat for the community and eco-system in Hanko.	- FMI. 2020b. Finnish Meteorological Institute (FMI) [WWW Document]. URL: www.fmi.fi
13	**Ocean/Coastal Parameters**	The algal bloom have become increasingly frequent in recent years as a result of the increase in temperature in Baltic and Finnish coastal waters, as well as the increase of pollution in these seas which provide ample nutrients for these organisms.	- FMI. 2020a. Finnish Meteorological Institute (FMI) - Marine research. [WWW Document]. URL: https://en.ilmatieteenlaitos.fi/marine-research
14	**Habitats**	Habitats for fish, birds (e.g. gull), Baltic clam, grey seal, sand dune, and many more.	- FMI. 2020a. Finnish Meteorological Institute (FMI) - Marine research. [WWW Document]. URL: https://en.ilmatieteenlaitos.fi/marine-research
15	**Groundwater salinization**	48.5 km^2, due to the potential increase of future sea level rise and storm surges, water intake wells could be moved inland in the higher elevation. Low impacts of salininity on water quality due to low saliniity of the Baltic Sea.	- Luoma, S., Klein, J. & Backman, B. 2013. Climate change and groundwater: Impacts and Adaptation in shallow coastal aquifer in Hanko, south Finland. In: Schmidt-Thomé, P. & Klein, J. (eds) Climate Change Adaptation in Practice – From Strategy Development to Implementation. Wiley-Blackwell, 137−155.<br>- Luoma, S. & Okkonen, J. 2014. Impacts of Future Climate Change and Baltic Sea Level Rise on Groundwater Recharge, Groundwater Levels, and Surface Leakage in the Hanko Aquifer in Southern Finland. Water 6:3671-3700, doi:10.3390/w6123671<br>- Luoma, S., Okkonen, J., Korkka-Niemi, K., Hendriksson, N. & Backman, B. 2015. Confronting vicinity of the surface water and sea shore in a shallow glaciogenic aquifer in southern Finland, Hydrol. Earth Syst. Sci. 19:1353-1370, doi:10.5194/hess-19-1353-2015<br>- Luoma, S., Okkonen, J. & Korkka-Niemi, K. 2017. Comparison of the AVI, modified SINTACS and GALDIT vulnerability methods under future climate-change scenarios for a shallow low-lying coastal aquifer in southern Finland. Hydrogeology J. 25: 203. doi.org/10.1007/s10040-016-1471-2
16	**Base Rock**	Pre-Cambrian crystalline bedrock with locally fracture zones that could act as conduit for the salinization from the seawater.	- GTK. 2020. Bedrock map of Finland at scale 1: 1 000 000. Geological Survey of Finland. Espoo. Finland.
17	**Other non-coastal natural hazards**	No volcanoes or active earthquakes. Heatwaves could possible take place during the extreme temperature during summer.	- FMI. 2020b. Finnish Meteorological Institute (FMI) [WWW Document]. URL: www.fmi.fi

Hanko, Finland

Table 2: Typology to assess exposure and vulnerability of a coastal locality

#	Exposure and Vulnerability Elements	Indicators / Metrics	Sources
18	**Population**	8517 (31.12.2017), 779, no exact number available, but the numbers of tourist during peak season (summer) increase more than double of normal time.	- Hanko. 2020. Information of the Hanko City. [WWW Document]. URL: www.hanko.fi<br>- Statistics Finland. 2020a. Population and Population projection of Finland. [WWW Document]. URL: https://www.stat.fi/til/vrm_en.html
19	**Future Population Change**	Decreasing, predicted approx.6119 in 2040.	- Hanko. 2020. Information of the Hanko City. [WWW Document]. URL: www.hanko.fi<br>- Statistics Finland. 2020a. Population and Population projection of Finland. [WWW Document]. URL: https://www.stat.fi/til/vrm_en.html
20	**Historic coastal and/or fluvial flooding**	The highest stormsurge took place in January 2005 with the maximum sea level of +132 cm. Water level increased within 8 hrs and lasted 1.5 days (36hrs), buildings and roads in the low-lying areas along the beaches and coastline, harbour/ports.	- FMI. 2020b. Finnish Meteorological Institute (FMI) [WWW Document]. URL: www.fmi.fi<br>- Hanko. 2020. Information of the Hanko City. [WWW Document]. URL: www.hanko.fi
21	**Human Development Index (national)**	0.92 (2017, national)	- UNDP. 2020. Human Development Report: Finland. The United Nations Development Programme. [WWW Document]. URL: http://hdr.undp.org/en/countries/profiles/FIN
22	**GNP/capita (national)**	GNI of 43,780 (2016 PPP $, national)	- Eurostat. 2020. The European statistical data. [WWW Document]. URL: https://ec.europa.eu/eurostat<br>- Statistics Finland. 2020b. Annual national accounts. [WWW Document]. URL: http://www.stat.fi/til/vtp/index_en.html<br>- World Bank. 2020. The World Bank Data. [WWW Document]. URL: http://data.worldbank.org/indicator/NY.GNP.PCAP.PP.CD?view=map
23	**Proportion of national population that is coastal**	0.15% (8,517 of total Finnish population 5,513,130 in 31.12.2017)	- Statistics Finland. 2020a. Population and Population projection of Finland. [WWW Document]. URL: https://www.stat.fi/til/vrm_en.html
24	**Governance**	Municipality, Federal or national ministry or agency	- Finnish Government. 2020. Information of the Finnish Government. [WWW Document]. URL: https://valtioneuvosto.fi/en/government
25	**Relationships to larger governmental entities**	Municipalities of Uusimaa, Uusimaa Region	- Finnish Government. 2020. Information of the Finnish Government. [WWW Document]. URL: https://valtioneuvosto.fi/en/government
26	**Relationships to international entities**	EU, national aid agencies, environmental nonprofit groups, NGO	- Finnish Government. 2020. Information of the Finnish Government. [WWW Document]. URL: https://valtioneuvosto.fi/en/government
27	**Built Infrastructure**	Ports, transport infrastructure, water intake wells, waste water treatment plants, health center, airport, high-way, railway.	- Hanko. 2020. Information of the Hanko City. [WWW Document]. URL: www.hanko.fi
28	**Natural Capital**	There is still large uncertainty of climate impact on changes of ecosystem, species and habitats, where the climate change could be benefit or disadvantage. Reducing fish stocks, for example, can cause by many factors, not only climate change.	- FMI. 2020b. Finnish Meteorological Institute (FMI) [WWW Document]. URL: www.fmi.fi<br>- Hanko. 2020. Information of the Hanko City. [WWW Document]. URL: www.hanko.fi<br>- Luke. 2020. Natural resources of Finland. [WWW Document]. URL: https://www.luke.fi/en/natural-resources/<br>- NLS. 2020. National Land Survey of Finland (NLS). [WWW Document]. URL: https://www.maanmittauslaitos.fi/en
29	**Available geographic/GIS data**	Many kinds of maps and spatial data, e.g. land survey, topography, GIS, registeration	- NLS. 2020. National Land Survey of Finland (NLS). [WWW Document]. URL: https://www.maanmittauslaitos.fi/en
30	**Minority status**	Bilingual town of Hanko consists of Finnish speaking 53.5%, Swedish speaking 42.8% and diversity of foreigners and religion, 3.7 % of total poppulation 8517 (31.12.2017).	- Hanko. 2020. Information of the Hanko City. [WWW Document]. URL: www.hanko.fi<br>- Statistics Finland. 2020a. Population and Population projection of Finland. [WWW Document]. URL: https://www.stat.fi/til/vrm_en.html
31	**Historical areas**	The whole Hanko Cape is an historic area since the late 13th century through the Winter War 1940.	- Hanko. 2020. Information of the Hanko City. [WWW Document]. URL: www.hanko.fi
32	**Environmental areas**	4491 ha (land-area 1133 ha, seawater-area 3358), Natural protected areas	- Hanko. 2020. Information of the Hanko City. [WWW Document]. URL: www.hanko.fi<br>- Ministry of the Environment. 2020. The protected nature areas in Finland. [WWW Document]. URL: http://www.environment.fi/en- US/Nature/Protected_areas
33	**Cultural areas**	Variety of cultures in Hanko-a bilingual (Finnish and Swedish) port town, coastline, archipelago.	- Hanko. 2020. Information of the Hanko City. [WWW Document]. URL: www.hanko.fi
34	**Tourism areas**	Different kinds of tourist sites from historic places to nautre areas, e.g.30 km sandy beaches, 4490 ha archipelago with 90 small islands and isles, museums, harbours	- Hanko. 2020. Information of the Hanko City. [WWW Document]. URL: www.hanko.fi
