# Supplementary material for: Challenges to climate change adaptation in coastal small towns: Examples from Ghana, Uruguay, Finland, Denmark, and Alaska
Source: Ocean Coast Manag. 2021 Oct 15;212:105787. doi: 10.1016/j.ocecoaman.2021.105787 (PMC10644629; doi:10.1016/j.ocecoaman.2021.105787)
Supplement: Multimedia component 3 [file mmc3.pdf]

Climate Change Adaptation in Kiyú, San José, Uruguay

Gustavo J. Nagy[1], Ofelia Gutiérrez[1], Daniel Panario[1], Inti Carro[2], Leo Seijo[3], Carolina Segura[4], José E. Verocai[1]

[1]Instituto de Ecología y Ciencias Ambientales, Facultad de Ciencias, Universidad de la República, Iguá 4225, CP 11400, Montevideo, Uruguay

[2]Dirección Nacional de Cambio Climático, Ministerio de Ambiente, Uruguay

[3]Programa de Desarrollo y Gestión Subnacional, Oficina de Planeamiento y Presupuesto, Presidencia de la República, Edificio Libertad, Montevideo, Uruguay

[4]Dirección Nacional de Biodiversidad y Servicios Ecosistémicos, Ministerio de Ambiente, Uruguay

Abstract

The Climate Adaptation (CA) model in Uruguay lies on participatory processes, scientific research and capacity building of institutions and local stakeholders, so that knowledge can be integrated into the design and application of policies and collective action. The case of Kiyú Beach, at the Rio de la Plata's tidal river estuary, shows the exposure to sea-level rise and extreme events, and the vulnerability of sandy beaches, and the touristic infrastructure. The CA lies on two pillars i) vulnerability reduction assessment (VRA) and ii) ecosystem-based adaptation (EbA), plus a beach profiling monitoring. The VRA assesses the community perceptions of risk, barriers and opportunities to adapt and develop a Community Based Adaptation (CBA) to arrest erosion. The implementation of EbA measures (e.g. the construction of storm drains and placement of sand-captor fences) was a valuable input for the national coastal adaptation plan. The main successes were the rapid recovery of sandy beach and dunes and socio-institutional learning process and strengthening.

1.0 Introduction

Coastal regions are increasingly vulnerable to climate stressors (CS) and are impacted by them regardless of their socioeconomic development, mainly because of the mostly uncontrollable external drivers, namely sea-level rise (SLR) and storm surges, as well as non-climate stressors (NCS), which are modifiable in the short-term (Leal Filho et al., 2018).

Climate vulnerability (CV) is herein the propensity to be adversely affected by hazards; it encompasses geographical and climatic exposure (E), sensitivity (S), and inversely by adaptive capacity (AC) (IPCC, 2007; U.S. Climate Resilience Toolkit, 2018).

$$[CV= (E + S – AC)].$$

Climate resilience (CR) is a key factor of the actor-based decision-making process of adaptation of socio-ecological system (SES) to climate change (Folke, 2006) and extreme events such as storm surges and heavy erosive precipitation in coastal areas (Carro et al., 2018). CR is equally understood as having both natural (system-based) and human (actor-based) components (Carro et al., 2018; Leal Filho et al., 2018) which may be regarded as an orientation of decision-making rather than as a policy outcome with the process linking resources (adaptive capacities) to outcomes (adaptation) (Norris et al., 2008). Climate Adaptation (CA) is herein: “Monitoring change and undertaking deliberate and considered adjustment actions and reinforcement strategies in natural and human systems to avoid, cope, or reduce harms and losses of climate change and variability, and their effects” (IPCC, 2007; UKCIP, 2014).

Maintaining coastal environments resilience involves analysing the capacity to generate the necessary conditions for maintaining ecosystem functions that support the services provided by beaches (Flood and Schechtman, 2014) and other coastal landscapes such as dunes, bluffs and their associated vegetation (Gutiérrez et al., 2015).

Because of the changing climate on coastal areas of South America, Uruguay in particular, adapting to "climate threats" along with mismanagement pressures will be an ongoing challenge into the future (Villamizar et al., 2017). The CA model in Uruguay lies on participatory processes based on scientific research and capacity building of institutions and local stakeholders, so that knowledge can be integrated into the design and application of policies and collective action (Nagy et al., 2015, 2014b).

2.0 Background

Two successive extreme storm surges in September and October 2012 impacted Kiyú Beach, San José, Uruguay, at the Rio de la Plata's tidal river estuary coast (Figure 1) highlighting the coastal exposure to extreme events. This micro-tidal coastline is characterised by erosive bluffs, sandy beaches and dunes, vulnerable to storm surges, Sea-level Rise (SLR) and flooding associated with riverine floods (triggered by El Niño events). The storm surges eroded the sandy beach, dune, and bluffs, while fallen trees broke the bluff; a touristic site, the "Parador Chico" Hostel, and coastal roads were also damaged.

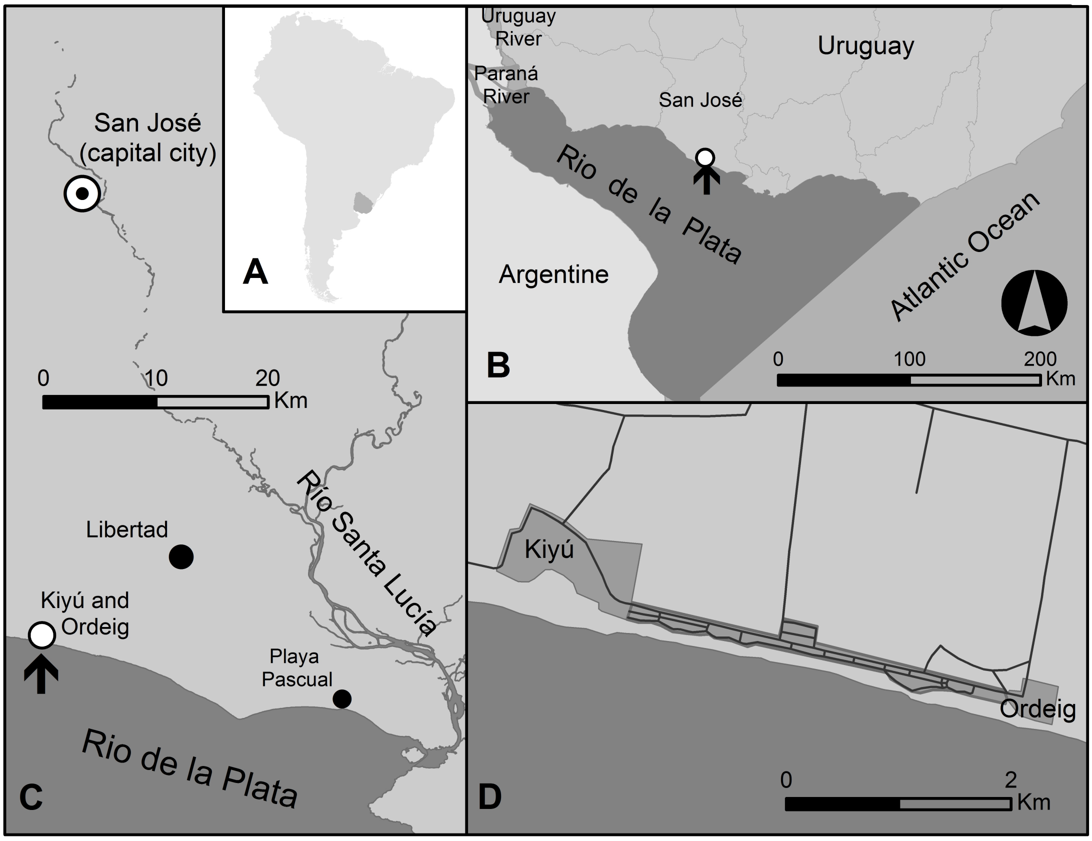

Figure 1: Study site. A) South America. B) Rio de la Plata river estuary. The black arrow indicates the pilot coastal adaptation site at San José: Kiyú. C) Kiyú and its surroundings. D) Kiyú - Ordeig in detail.

An assessment of climate risks carried out by a Global Environment Facility (GEF) project considered the impacts of extreme storms under an SLR scenario (+ 0.2 m) to the coastal landscapes and private property as high to very high, which is a considerable concern for the national and sub-national authorities and local stakeholders as the primary revenue from tourism was threatened. Therefore, the extreme events were a window of opportunity for planning adaptation measures at Kiyú Beach. A community-level adaptation experience was developed from 2013 to 2015 through an agreement between the National Directorate of the Environment (DINAMA), the subnational government of San José (GDSJ), local-level authorities (Municipality of Libertad), and local stakeholders (Carro et al., 2018). The coastal management experience was based on two pillars i) vulnerability reduction assessment (VRA) and ii) ecosystem-based adaptation (EbA), plus a beach profiling monitoring.

The VRA (Crane Droesch et al., 2008) is designed to measure community-level adaptive capacity to climate change and variability in Community Based Adaptation (CBA) monitoring and evaluation activities. It is a relative measure of change against a pre-project baseline (before-after implementing measures). VRA was conducted according to Nagy et al. (2014b) from 2011 to 2014 to assess decision-makers and stakeholders' vulnerability and risk

perception, as well as barriers and opportunities to implement adaptation. The VRAs consisted of the following four stages:

1) semi-structured and in-depth interviews with different groups of stakeholders;
2) focus group discussions;
3) a multiple question matrix was filled with qualitative comments and a numerical assessment (a Likert 1-5 scale) measured through corresponding open-ended, perception-based questions, which in turn aggregate to serve as indicators of adaptive capacity was used to implementing adaptation; and
4) a dialogue between participants and experts was held to achieve a collectively agreed value for each question and oral comments (Carro et al., 2018; Nagy and Gutiérrez, 2018).

The results of VRAs were expressed as the average of a discrete 1-5 Likert-scale (e.g., 3.1) and summarised as follows:

- The erosion of sandy beaches, dunes, and bluffs –due to SLR, wind-induced flooding, and mismanagement issues– was perceived as a climate threat or impact by most participants (Carro et al., 2018; Nagy and Gutiérrez, 2018)(Leal Filho et al., 2018).
- Because SLR trend was 0.1-0.2 m (Nagy et al., 2007; Verocai et al., 2016), most participants did not perceive them as an impact but as a threat (Nagy and Gutiérrez, 2018).
- Based on stakeholders perceptions of threats and risks (Nagy et al., 2014b, 2014a; Verocai et al., 2015), increasing SLR of 0.1, 0.2, 0.3 and 0.4 were fixed as being very low (1), low (2), moderate (3) and high (4) respectively. Storm surges were placed as the main threat; while SLR >0.3 m, extreme rainfall, and southern wind changes (SE or SW), were set at the second, third and fourth threats (Nagy and Gutiérrez, 2018).

The EbA experience was a valuable input for the DINAMA's national coastal adaptation plan (Coastal NAPA), Coastal Management Division, and Climate Change Division. Some successes of the Kiyú experience were the:

- Rapid recovery of sandy beach and dunes after the implementation of measures.
- Socio-institutional learning process and strengthening.
- Capacity building in soft-ecological actions (green infrastructure) to sustain the process.

- Subnational and local governments budget for EbA activities.
- Mainstreaming of EbA approach at the scale of the sub-national and local governments.
- Creation of the Departmental Office of Climate Change (DOCC) in 2015 allowed reaching cross-sector effective coastal management partnership with the national government and stakeholders, beyond the specific climate adaptation goals.

The main recommendations were:

- Further actions need to be implemented to achieve the desired state of Kiyú Beach, e.g. to prevent storm drains continue wetting the recovered sand,
- the displacement of the touristic infrastructure far away from the bluffs, and
- to organise a fourth VRA to evaluate outcomes.

3.0 Materials and Methods

The data used for this case study are based on i) Previous literature from the authors (Carro et al., 2018; Gutiérrez et al., 2016, 2015; Nagy et al., 2015; Nagy and Gutiérrez, 2018); ii) beach profiles, storm surges, and water-level (2015-2018); iii) a field survey; and iv) a VRA workshop held with the authorities, municipal managers and stakeholders in October 2018 (Table 1).

The monitoring of the recovery actions was carried out employing the indicator "Beach profile" (Carro et al., 2018), which consists of the measurement of slope variation in a transect from the primary dune to the high berm, useful to monitor changes in coastal configuration over time.

Table 1: Stakeholders' perceptions of threats and impacts, vulnerability and adaptive capacity (V and AC).

Year	Threats	V / AC	Comments
1. Baseline (2011)	Extreme events. Erosion	4.2 / 1.5	Lack of local adaptive capacity
2. Before EbA implementation (2013)	Windstorms. Floodings. Beach and bluff erosion	2.5 / 2.1	Lack of adaptive capacity and information regarding impacts and possible adaptation measures / Confidence in local commitment and local authorities.
3. After EbA implementation (2014)	Windstorms. Rain-storms. SLR, increased extremes	1.8 / 3.3	Increased perception of threats after the damaging rainstorm in early 2014. Increased confidence in adaptive capacity.
4. Monitoring and Evaluation (2018)	Windstorms. Erosive rain-storms.	1.8 / 3.3	Decreased perception of threats and vulnerability; similar adaptive capacity (compared with 2014). Future scenarios and adaptation processes are understood. Increased need for new storm-drains.

4.0 Results and Discussion

Figure 2 shows a sustained increase in the beach slope, accretion and building of the dune and the high berm between 2013 and 2018, after the recovery actions in the coastal ecosystem carried out in 2013, including the construction of storm drains ("gutters") and placement of sand-captor fences for dune regeneration. During 2014 and 2015, only dune regeneration actions were carried out. As of 2016, the process of sand accumulation continued, which was facilitated by the spontaneous development of psammophile vegetation, although no interventions of any kind were carried out until 2018.

The evolution of the beach profiles from 2013 to 2018 showed an increase (Figure 2) in the average height (≥1 m) and volume (≥46%) of sand, a well-developed high water level berm (sensu Boak and Turner, 2005), and a more massive vegetated dune. The dune cannot further develop here because of a touristic building ("Parador Chico" Hostel) and a pedestrian path; however, a vegetated dune has grown on both sides of the hostel since 2016. Notably, none of the six storm surges – some of which have been similar to the ones of 2012 – and high-intensity rains that occurred since 2013 put the coastal infrastructure at risk in the area where EbA actions were carried out.

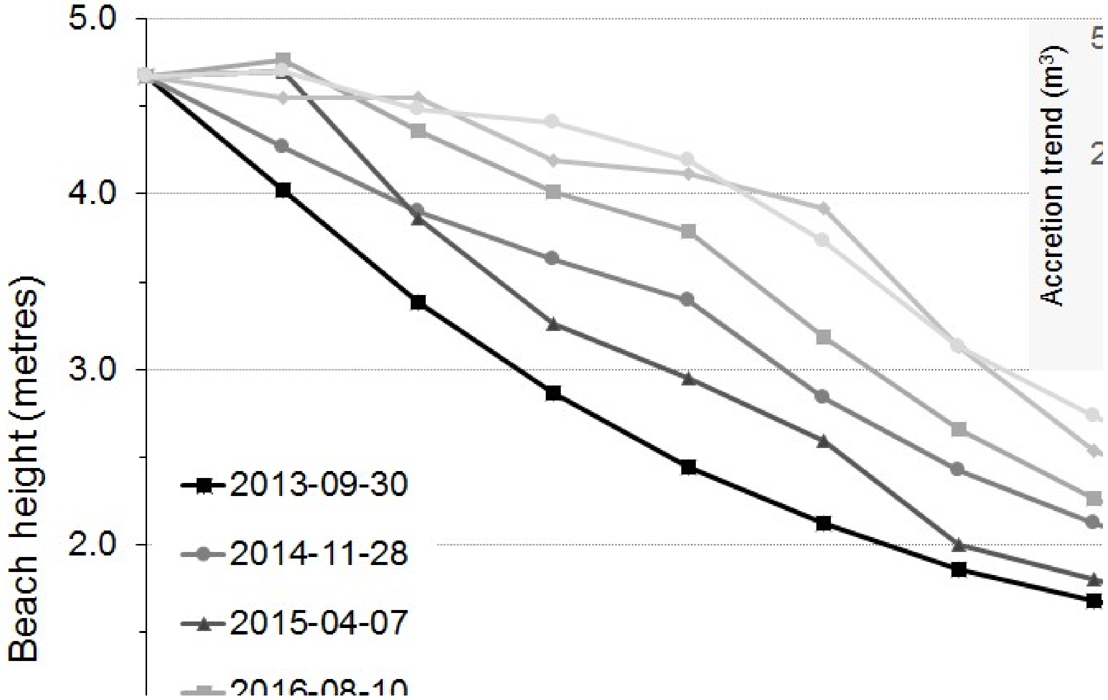

Figure 2: Kiyú Beach selected profiles from 2013 to 2018. The profiles show 44 m of the beach from the high berm to the dune. The accretion trend (from 0 to 46 m^3) for a 1m-wide profile section is shown in the small box (upper right).

As of October 2018, all participants believe that the only SLR is a (long-term) critical issue because they are confident that the green infrastructure and recovered berm and dune are resilient to extreme events. Beach erosion and damages to infrastructure were placed as the main three impacts, but only erosion is perceived as a potential impact, mainly due to heavy rains and channelling. Therefore, priority is put on the construction of new gutters.

The cost of the soft-ecological recovery actions is estimated from yearly beach maintenance unit costs (MUC) before 2013. The MUCs have been five, one for the placement of green infrastructure and four for the construction of storm-drains, in 2013, one in 2014 and 2015, and zero since 2016, so as the actions have resulted both cost-effective and efficient.

In regards to the recommendations made by Carro et al. (2018), the desired state of Kiyú landscape and ecosystem was almost achieved by 2018, except i) the complete substitution of tall trees and ii) the displacement of the touristic infrastructure ("Parador Chico" Hostel and pedestrian path) far away from the bluffs. The latter is no more a priority due to the achieved increase in resilience. A recent success is the replication of the community-level EbA experience developed at Kiyú Beach in three places as of 2018, two of them in San José: i) Kiyú and Ordeig and ii) Playa Pascual (see Figure. 1). The both are funded by a GEF Small Grants Programme (SGP) and implemented by local stakeholders supported by the Subnational Government of San José (GDSJ) and the National Directorate of the

Environment (DINAMA); the former is developed by people that have participated in the Kiyú experience, plans to place 1,200 m of sand captors in 2019.

5.0 Conclusions

The erosive processes impacting the coastal system and touristic infrastructure was arrested through a successful Ecosystem-based Adaptation approach developed through a management arrangement involving the community network, local, subnational and national-level stakeholders and authorities.

The beach height and volume have increased, particularly the high berm, whereas the primary dune has developed aside from the "Parador Chico Hostel". The improvement was rapid from 2013 to 2017, and some stabilisation is observed as of 2018.

The Neighborhood Commission generated a restoration project and became a co-executor of it and not only as a participant (as happened at the beginning of the process).

The Kiyú Beach experience is being replicated, particularly at San José, through GEF Small Grants Programme initiatives supported by both the National and Subnational Governments.

6.0 References

Boak, E.H., Turner, I.L., 2005. Shoreline Definition and Detection: A Review. J. Coast. Res. 21, 688–703. doi:10.2112/03-0071.1

Carro, I., Seijo, L., Nagy, G.J., Lagos, X., Gutiérrez, O., 2018. Building capacity on ecosystem-based adaptation strategy to cope with extreme events and sea-level rise on the Uruguayan coast. Int. J. Clim. Chang. Strateg. Manag. 10, 504–522. doi:10.1108/IJCCSM-07-2017-0149

Crane Droesch, A., Gaseb, N., Kurukulasuriya, P., Mershon, A., Moussa, K.M., Rankine, D., Santos, A., 2008. A Guide to the Vulnerability Reduction Assessment, UNDP Working Paper. Community-Based Adaptation Programme, United Nations Development Programme (UNDP), New York, U.S.A. URL http://www.seachangecop.org/files/documents/2008_12_CBA_Vulnerability_Reducation_Assessment_Guide.pdf (accessed 7.6.17).

Flood, S., Schechtman, J., 2014. The rise of resilience: Evolution of a new concept in coastal planning in Ireland and the US. Ocean Coast. Manag. 102, 19–31. doi:10.1016/j.ocecoaman.2014.08.015

Folke, C., 2006. Resilience: The emergence of a perspective for social–ecological systems analyses. Glob. Environ. Chang. 16, 253–267. doi:10.1016/J.GLOENVCHA.2006.04.002

Gutiérrez, O., Panario, D., Nagy, G.J., Bidegain, M., Montes, C., 2016. Climate teleconnections and indicators of coastal systems response. Ocean Coast Manag 122:64–76. doi:10.1016/j.ocecoaman.2016.01.009

Gutiérrez, O., Panario, D., Nagy, G.J., Piñeiro, G., Montes, C., 2015. Long-term morphological evolution of urban pocket beaches in Montevideo (Uruguay): impacts of coastal interventions and links to climate forcing. J. Integr. Coast. Zo. Manag. - Rev. Gestão Costeira Integr. 15, 467–484. doi:10.5894/rgci553

IPCC, 2007. Climate Change 2007: Impacts, adaptation and vulnerability. Contribution of Working Group II to the Fourth Assessment Report of the Intergovernmental Panel on Climate Change. Cambridge University Press, Cambridge, United Kingdom.

Leal Filho, W., Modesto, F., Nagy, G.J., Saroar, M.M., YannickToamukum, N., Ha'apio, M., 2018. Fostering coastal resilience to climate change vulnerability in Bangladesh, Brazil, Cameroon, and Uruguay: a cross-country comparison. Mitig. Adapt. Strateg. Glob. Chang. 23, 579–602. doi:10.1007/s11027-017-9750-3

Nagy, G.J., Gómez-Erache, M., Fernández, V., 2007. El aumento del nivel del mar en la costa uruguaya del Río de la Plata. Tendencias, vulnerabilidades y medidas para la adaptación. Medio Ambient. y Urban. (ISSN 0326-7857) 67, 77–93.

Nagy, G.J., Gómez-Erache, M., Kay, R., 2015. A risk-based and participatory approach to assessing climate vulnerability and improving governance in coastal Uruguay, in: Glavovic, B., Kelly, M., Kay, R., Travers, A. (Eds.), Climate Change and the Coast Building Resilient Communities. CRC Press, Boca Raton, Florida, U.S.A., pp. 357–378. doi:10.1201/b18053-23

Nagy, G.J., Gutiérrez, O., 2018. Scenario planning toward climate adaptation: The Uruguayan coast, in: Leal Filho, W., Esteves de Freitas, L. (Eds.), Climate Change Adaptation in Latin America. Managing Vulnerability, Fostering Resilience, Climate Change Management Series. Springer, Cham, Switzerland, pp. 457–476. doi:10.1007/978-3-319-56946-8_28

Nagy, G.J., Muñoz, N., Verocai, J.E., Bidegain, M., Seijo, L., 2014a. Adjusting to current climate threats and building alternative future scenarios for the Rio de la Plata coast and estuarine front, Uruguay. J. Integr. Coast. Zo. Manag. - Rev. Gestão Costeira Integr. 14, 553–568. doi:10.5894/rgci472

Nagy, G.J., Seijo, L., Verocai, J.E., Bidegain, M., 2014b. Stakeholders' climate perception and adaptation in coastal Uruguay. Int. J. Clim. Chang. Strateg. Manag. 6, 63–84. doi:10.1108/IJCCSM-03-2013-0035

Norris, F.H., Stevens, S.P., Pfefferbaum, B., Wyche, K.F., Pfefferbaum, R.L., 2008. Community resilience as a metaphor, theory, set of capacities, and strategy for disaster readiness. Am. J. Community Psychol. 41, 127–150. doi:10.1007/s10464-007-9156-6

U.S. Climate Resilience Toolkit, 2018. Glossary [WWW Document]. URL https://toolkit.climate.gov/content/glossary (accessed 7.6.18).

UKCIP, 2014. Glossary [WWW Document]. United Kingdom Clim. Impacts Program. URL http://www.ukcip.org.uk/glossary/ (accessed 6.15.16).

Verocai, J.E., Gómez-Erache, M., Nagy, G.J., Bidegain, M., 2015. Addressing climate extremes in Coastal Management: The case of the Uruguayan coast of the Rio de la Plata System. J. Integr. Coast. Zo. Manag. - Rev. Gestão Costeira Integr. 15, 91–107. doi:10.5894/rgci555

Verocai, J.E., Nagy, G.J., Bidegain, M., 2016. Sea-level trends along freshwater and seawater mixing in the Uruguayan Rio de la Plata estuary and Atlantic Ocean coast. Int. J. Mar. Sci. 6, 1–18. doi:10.5376/ijms.2016.06.0007

Villamizar, A., Gutiérrez, M.E., Nagy, G.J., Caffera, R.M., Leal Filho, W., 2017. Climate adaptation in South America with emphasis in coastal areas: the state-of-the-art and case studies from Venezuela and Uruguay. Clim. Dev. 9, 364–382. doi:10.1080/17565529.2016.1146120

Kiyú, Uruguay

TABLE 1: Typology to assess the hazards and susceptibility of a coastal locality

#	Hazard and Susceptibility Elements	Indicators / Metrics	Sources
1	**Settlement location**	34°41′48″S 56°44′53″; km from present and future (2050) shoreline; 0.4/0.2 m above present/future mean sea-level (based on 1990 levels).	- The authors
2	**Köppen–Geiger climate classification system**	16.8°C (1961-90; InuMet, 2018); cfa (prevailing) and cfb (coastal); not expected in the mid-term, notwithstanding the observed increase in subtropical indicators over the last 3-5 decades (e.g. increased precipitations and a decrease in the number of freezing days, Nagy et al., 2016). Mean temperature has increased > 0.5º C from 1961 to 2014, reaching ≈17.3 ºC. Increase in calm days and winds NE and decrease in winds SSW and SW. Changes in the annual distribution of rainfall and storm frequency, particularly during spring and summer months, are predicted (Nagy et al., 2016; Gutiérrez et al., 2016).	- Gutiérrez, O., Panario, D., Nagy, G.J., Bidegain, M., Montes, C., 2016. Climate teleconnections and indicators of coastal systems response. Ocean & Coastal Management 122, 64–76. https://doi.org/10.1016/j.ocecoaman.2016.01.009<br>- InuMet, 2018. Series 1961-1990 [WWW Document]. Uruguayan Institute of Meteorology, Montevideo, Uruguay.<br>- Nagy, G.J., Bidegain, M., Verocai, J.E., de los Santos, B., 2016. Escenarios climáticos futuros sobre Uruguay. Ministerio de Vivienda, Ordenamiento territorial y Medio Ambiente (MVOTMA) División de Cambio Climático (DCC), Montevideo.
3	**Isostatic rebound**	It is estimated as being in the order of millimetres (based on data from Codignotto et al., 2018) for the Argentinean coast of the RdlP estuary.	- Codignotto, J.O., Kokot, R.R., Marcomini, S.C., 1992. Neotectonism and Sea-Level Changes in the Coastal Zone of Argentina. Journal of Coastal Research 8, 125–133. http://journals.fcla.edu/jcr/article/view/78661
4	**Subsidence**	It is estimated as being in the order of millimetres (based on data from Codignotto et al., 2018)	- Op cit.
5	**Local/regional mass density changes**	The rate of SLR was 1-2 mm/year over the past five decades, reaching yearly maximum associated with El Niño-related river flood events	- Verocai, J.E., Nagy, G.J., Bidegain, M., 2016. Sea-level trends along freshwater and seawater mixing in the Uruguayan Rio de la Plata estuary and Atlantic Ocean coast. Int. J. Mar. Sci. 6, 1–18. https://doi.org/10.5376/ijms.2016.06.0007
6	**Coastal erosion**	Yes. Ecosystem-based Adaptation approach; 1m/year (1,000 m over past 1,000 years) (Casciani and Musso, 1995).<br>Estimated at 1.7 m / cm from the application of the Bruun Rule for the adjacent Metropolitan Area of Montevideo, with an observed maximum of 4.7m / year (at the mouth of Pando River, located eastward) (Gutiérrez et al., 2015, 2005)	- Casciani, M., Musso, M., 1995. Caracterización sedimentológica y geomorfológica de la playa de Kiyú. Departamento de San José, Uruguay. Facultad de Ciencias, Montevideo. (Unpublished)<br>- Gutiérrez, O., Panario, D., 2005. Dinámica geomorfológica de la desembocadura del Arroyo Pando, Uruguay. Geografía histórica y SIG, análisis de tendencias naturales y efectos antrópicos sobre sistemas dinámicos. Xeográfica, Revista de Xeografía, Territorio e Medio Ambiente 5, 107–126.<br>- Gutiérrez, O., Panario, D., Nagy, G.J., Piñeiro, G., Montes, C., 2015. Long-term morphological evolution of urban pocket beaches in Montevideo (Uruguay): impacts of coastal interventions and links to climate forcing. J. Integr. Coast. Zo. Manag. - Rev. Gestão Costeira Integr. 15, 467–484. doi:10.5894/rgci553
7	**Slopes and angles on or near the shore**	Very variable slope depending on the existence or not of structural control (Gutiérrez and Panario, 2019). Structural control is also widespread in the Uruguayan coast, as a consequence of the inheritance of structures due to the retreat and advance of sea level (-130m 14000 to 17000 BP to + 5m in 6000 BP) (Bracco et al., 2014). Nearshore slope to the Paleocanal of the Uruguay River in the the Rio de la Plata estuary (along the Uruguyan coast) is a platform of marine abrasion whose slope is controlled by the resistance of the sediments (mostly Cenozoic)	- Bracco, R., Inda, H., del Puerto, L., Capdepont, I., Panario, D., Castiñeira, C., García-Rodríguez, F. 2014. A reply to "Relative sea level during the Holocene in Uruguay." Palaeogeogr. Palaeoclimatol. Palaeoecol. 401, 166–170. https://doi.org/10.1016/j.palaeo.2013.10.012<br>- Gutiérrez, O., Panario, D., 2019. Caracterización y dinámica de la costa uruguaya, una revisión, in: Muniz, P., Conde, D., Venturini, N., Brugnoli, E. (Eds.), Ciencias Marino-Costeras en el Umbral del Siglo XXI, Desafíos en Latinoamérica y el Caribe. Editorial AGT Editorial S.A, México DF, México, p. 61–91
8	**Located in tropical or other storm zone**	Not applicable; 0 days above 26ºC	- The authors.
9	**Inland Rainfall**	The rainiest months in Kiyú are March and October (> 100 mm/month, 1961-90), increasingly in October over the last few decades. The area is drought-prone, particularly during summer months, particularly during La Niña years (e.g. January-February 1989, 2000, 2009)	- The authors.
10	**Inland rivers**	There are a few small rivers (e.g. Mauricio Creek) flowing to the RdlP close to Kiyú, but their floods do not reach the small town.	- The authors.

11	**Extent and likelihood of coastal and/or fluvial flooding**	Coastal flooding due to extreme increases in the accumulated river inflow to the Rio de la Plata estuary occur several times per decade, often related to strong and very strong El Niño events and increased rainfall in the Rio de la Plata basin (e.g. in 1983, 1997-98, 2009, 2015-16). More frequent and stronger rainfall and storms under a 10-20 cm SLR scenario may increase the occurrence of coastal flooding, with loss of landscape and substantial damages to infrastructure. Consequently, the water level increases by +10-20 cm in the tidal river and estuarine region of the RdlP during several weeks to a few months, thus increasing the vulnerability to storm surges. (Verocai et al., 2015). An earthquake in 1884 followed by a small tsunami was reported in a brief note in Nature (1884). Another earthquake in 1888 also produced a seiche (standing wave) that affected the estuary of the Rio de la Plata) (Sanchez-Bettucci et al., 2017).	- NATURE, 1884. Brief note on Montevideo earthquake. Nature 29, p. 437.<br>- Sánchez-Bettucci, L., Suárez, N., Campal, N., Curbelo, A., Latorres, E., Rodríguez, M., Castro, H., Loureiro, J., Arduin, F., Faraone, M., Pascale, A., Abelenda, E., Saltorio, J., Lefebre, R., 2017. Desarrollo de una red geofísica y geodésica nacional en Uruguay. Development of geophysical and geodetic network in Uruguay. Revista SUG (Sociedad Geológica del Uruguay) 20, 37–46.<br>- Verocai, J.E., Gómez-Erache, M., Nagy, G.J., Bidegain, M., 2015. Addressing climate extremes in Coastal Management: The case of the Uruguayan coast of the Rio de la Plata System. Journal of Integrated Coastal Zone Management - Revista de Gestão Costeira Integrada 15, 91–107. https://doi.org/10.5894/rgci555
12	**Air temperature**	Heatwaves are not frequent in the coastal areas of Uruguay, where temperature seldom reaches > 35º C. The average maximum temperature for the warmest month (January) is 30ºC, and the absolute maximum is 40ºC.	- The authors.
13	**Ocean/Coastal Parameters**	The tidal river estuary of the RdlP where Kiyú is located shows a highly variable pH (< 7 to > 8) (Nagy et al., 2002a). The algae bloom increased an order of magnitude in the past decade (Bonilla et al., 2015).	- Bonilla, S., Haakonsson, S., Somma, A., Gravier, A., Britos, A., Vidal, L., De León, L., Brena, B.M., Pírez, M., Piccini, C., Martínez de la Escalera, G., Chalar, G., González-Piana, M., Martigani, F., Aubriot, L., 2015. Cianobacterias y cianotoxinas en ecosistemas límnicos de Uruguay [Cyanobacteria and cyanotoxins in freshwaters of Uruguay]. INNOTEC 10, 9–22.<br>- Nagy, G.J., Gómez-Erache, M., López, C.H., Perdomo, A.C., 2002a. Distribution patterns of nutrients and symptoms of eutrophication in the Rio de la Plata River Estuary System. Hydrobiologia 475–476, 125–139. https://doi.org/10.1023/A:1020300906000
14	**Habitats**	The site is characterised by sandy beach and dunes, erosive bluffs, exotic trees (Eucalyptus) which have progressively been substituted by native shrubs since 2014 (Carro et al., 2018). There are no significant changes since the extraction from the regional aquifer (Raigón) is very low in the studied area due to the low density of population and because there is no industrial use	- Carro, I., Seijo, L., Nagy, G.J., Lagos, X., Gutiérrez, O., 2018. Building capacity on ecosystem-based adaptation strategy to cope with extreme events and sea-level rise on the Uruguayan coast. International Journal of Climate Change Strategies and Management 10, 504–522. https://doi.org/10.1108/IJCCSM-07-2017-0149
15	**Groundwater salinization**	There are no significant changes since the extraction from the regional aquifer (Raigón) is very low in the studied area due to the low population density and because there is no industrial use (The authors). However, there is a risk of salinization during prolonged very low river inflow to the Rio de la Plata estuary because of the up-ward salt intrusion from the outer estuary (e.g. December 1999-March 2000 during a strong La Niña event) (Nagy et al., 2002b)	- Nagy, G.J., Gómez-Erache, M., Perdomo, A.C., 2002b. Water Resources: Río de la Plata, in: Munn, T. (EIC), Douglas, I. (Eds.), Encyclopedia of Global Environmental Change. Volume 3, Causes and Consequences of Global Environmental Change. John Wiley & Sons Ltd., Chichester, pp. 723–726.
16	**Base Rock**	A predominance of Cenozoic sediments; the crystalline rock is thick, and there are no perforations to estimate its depth	- The authors.
17	**Other non-coastal natural hazards**	There are no volcanoes; the region is not susceptible to earthquakes or prolonged heatwaves.<br>Heavy rain-storms capable or eroding the beach, dune and cliffs because of water channelling (e.g. late January 2016) (Carro et al., 2018)	- The authors.<br>- Op cit.

Kiyú, Uruguay

Table 2: Typology to assess exposure and vulnerability of a coastal locality

#	Exposure and Vulnerability Elements	Indicators / Metrics	Sources
18	**Population**	500 inhabitants (as of 2018), reaching an average of 3,000 to 4,000 during summer months (January-February), peaking at ≥ 10,000 people during weekends. A moderate increase in inhabitants is expected over the near-future.	- The authors, based on interview with local authorities, and data from INE (2011).
19	**Future Population Change**	Minor changes expected in the mid-term.	The authors, based on interview with local authorities.
20	**Historic coastal and/or fluvial flooding**	The coastal Kiyú Beach built environment has been impacted by extreme coastal floodings, particularly when they occurred combined with heavy storms and rainfall (Carro et al., 2018)	- Carro, I., Seijo, L., Nagy, G.J., Lagos, X., Gutiérrez, O., 2018. Building capacity on ecosystem-based adaptation strategy to cope with extreme events and sea-level rise on the Uruguayan coast. International Journal of Climate Change Strategies and Management 10, 504–522. https://doi.org/10.1108/IJCCSM-07-2017-0149
21	**Human Development Index (national)**	0.804 (National) (2017). Very High (UNDP, 2018)	- UNDP, 2018. Human Development Indices and Indicators. 2018 Statistical Update. United Nations Development Programme, Washington DC. http://www.hdr.undp.org/sites/default/files/2018_human_development_statistical_update.pdf (accesed October 13, 2018)
22	**GNP/capita (probably national)**	$US (nominal) = 15,250; U (PPP) = 21,870 (World Bank, 2018)	- World Bank, 2018. Gross national income per capita 2017, Atlas method and PPP [WWW Document]. World Dev. Indic. database. http://databank.worldbank.org/data/download/GNIPC.pdf (accessed October 18, 2018).
23	**Proportion of national population that is coastal**	About 70% and 30% respectively (Nagy et al., 2015; Villamizar et al., 2017).	- Nagy, G.J., Gómez-Erache, M., Kay, R., 2015. A risk-based and participatory approach to assessing climate vulnerability and improving governance in coastal Uruguay, in: Glavovic, B., Kelly, M., Kay, R., Travers, A. (Eds.), Climate Change and the Coast Building Resilient Communities. CRC Press, Boca Raton, Florida, U.S.A., pp. 357–378. https://doi.org/10.1201/b18053-23<br>- Villamizar, A., Gutiérrez, M.E., Nagy, G.J., Caffera, R.M., Leal Filho, W., 2017. Climate adaptation in South America with emphasis in coastal areas: the state-of-the-art and case studies from Venezuela and Uruguay. Clim. Dev. 9, 364–382. https://doi.org/10.1080/17565529.2016.1146120
24	**Governance**	Full democracy (The Economist Intelligence Unit, 2017). Centralised (Unitary) Government; environmental matters are primarily managed at the national-level (Directorate of the Environment), and the sub-national level, with increasing local level and stakeholders participation (Nagy et al., 2014; Carro et al., 2018)	- Op cit.<br>- Nagy, G.J., Seijo, L., Verocai, J.E., Bidegain, M., 2014. Stakeholders' climate perception and adaptation in coastal Uruguay. International Journal of Climate Change Strategies and Management 6, 63–84. https://doi.org/10.1108/IJCCSM-03-2013-0035<br>- The Economist Intelligence Unit (2017). Democracy Index. https://infographics.economist.com/2018/DemocracyIndex. (consulted October 12, 2018)
25	**Relationships to larger governmental entities**	Kiyú Beach is a small settlement with a neighbour committee (local stakeholders) dependent on the municipal government of Libertad, which is part of the sub-national government of San José (GDSJ) (Carro et al., 2018). Local commitment and participation in implementing plans are increasing, which is made without (a significant) loss of decision-making centralised in the national and sub-national government level (Nagy et al., 2014). The EbA approaches and socio-institutional innovation made to arrest the degradation of the dune and bluffs have empowered locals (Carro et al., 2018).	- Op cit.<br>- Nagy, G.J., Seijo, L., Verocai, J.E., Bidegain, M., 2014. Stakeholders' climate perception and adaptation in coastal Uruguay. International Journal of Climate Change Strategies and Management 6, 63–84. https://doi.org/10.1108/IJCCSM-03-2013-0035
26	**Relationships to international entities**	The EbA experience developed from 2013 to 2015 was supported by the Global Environment Facility (GEF) Project "Implementing Adaptation measures to Climate Change in coastal areas of Uruguay". The EbA actions to be developed at Ordeig beach in 2019 are supported by a GEF Small Grants Programme (SGP).	- The authors, based on interview with local authorities
27	**Built Infrastructure**	There are a few hundred buildings, mostly summer houses, and a basic transport infrastructure; there are not airport or wastewater treatment facilities close to Kiyú Beach.	- The authors

28	**Natural Capital**	The original vegetation at the site where there were bluffs were shrubs and small trees, while where there was no cliff, or it was shallow, there was herbaceous vegetation interspersed with patches of grassland and shrubs. This native vegetation was gradually replaced by afforestation with exotic species (pines and eucalyptus) and urbanisation. According to historical records (Giuffra, 1935), the coastal dunes reached the order of 5 meters in height. There is no significant loss of income reported.	- Giuffra, E.S., 1935. La República Del Uruguay. A. Monteverde y Cia., Montevideo.
29	**Available geographic/GIS data**	According to the last census (INE, 2011) there are 790 homes, 178 occupied and 612 vacant (probably tourist residences) in the tourist area of Kiyú - Ordeig. The permanent population was 423 people, and in the summer season about 3,000 people spend the night, and they could reach 10,000 as day visitors.	- INE, 2011. 8vo. Censo de Población y Vivienda. Instituto Nacional de Estadística. http://www.ine.gub.uy/web/guest/censos-2011 (accesed October 6, 2018)
30	**Minority status**	Not applicable in the studied area.	- The authors
31	**Historical areas**	In the dunes there are pre-Hispanic archaeological sites with ceramics and lithic material (DINOT, 2011).	- DINOT, 2011. Plan local de ordenamiento territorial de Kiyú y sus vecindades. Departamento de San José, República Oriental del Uruguay. http://www.imsj.gub.uy/portal15/pdf/APENDICE%20I.pdf (accesed October 10, 2018)
32	**Environmental areas**	Kiyú is a unique "Beach - Country" small settelement, with notable landscape, ecological and cultural values. It is adjacent to the metropolitan area of Montevideo (DINOT, 2011), and in relative proximity to the metropolitan area of Buenos Aires (Argentina).	- Op cit.
33	**Cultural areas**	There is a strong cultural heritage referred to the rural tradition and archaeological sites (Carro et al., 2018).	- Op cit.
34	**Tourism areas**	It is subdivided into a coastal zone, a shallow urbanized area towards the mainland (160 hectares) and border of agricultural-livestock fields (DINOT, 2011).	- Op cit.
