# Supplementary material for: Challenges to climate change adaptation in coastal small towns: Examples from Ghana, Uruguay, Finland, Denmark, and Alaska
Source: Ocean Coast Manag. 2021 Oct 15;212:105787. doi: 10.1016/j.ocecoaman.2021.105787 (PMC10644629; doi:10.1016/j.ocecoaman.2021.105787)
Supplement: Multimedia component 4 [file mmc4.pdf]

A case study of coastal management within Lemvig, Denmark

James M. Fitton[1*]

[1]MaREI, University College Cork, Ireland

Abstract

Denmark has a long coastline, and as a result is regularly impacted by storm surges and flooding. Lemving, a town in west Jutland, has previously been flooded, and as a result an innovative multipurpose sea wall has been install at the harbour front. While this currently offers protection, sea level rise will continue to make flooding a major risk in the future. Lemvig is part of the Coast To Coast Climate Challenge, a large project that includes many stakeholders in central Denmark and beyond. This will allow the town to leverage expertise and knowledge in order to more easily plan for the future risks of climate change.

1.0 Introduction

Denmark has an extensive coastline (7,300 km) and it is estimated that 40% of the Danish population lives within 3 km of the coast (Sørenson, 2013, p. 96). The coast includes numerous urban areas, holiday homes, and recreational areas, but substantial lengths remain natural (Kappel, Rasmussen, & Waneck, 2010). Lemvig is a town of 6,978 people, located with Lemvig Municipality (20,015 population in total), western Jutland, Denmark (Figure 1). The municipality shows negative population growth (one of the highest in the country) and more than 50% of the residents are aged over 40. While the overall population growth is negative, the predictions for population growth amongst those age 65 and above are increasing (Lemvig Kommune, 2017)

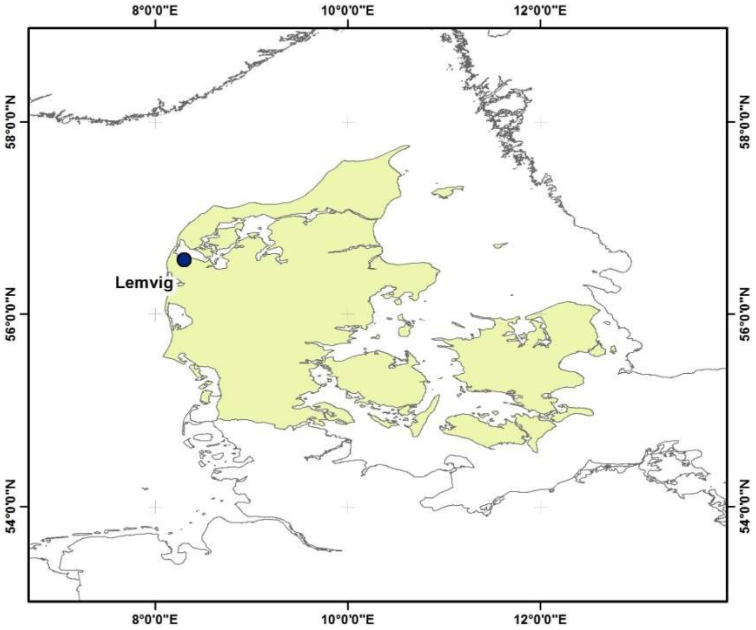

Figure 1: The Jutland Peninsula in Denmark, showing the location of Lemvig. Denmark.

*Corresponding Author: james.fitton@ucc.ie

Lemvig is situated on the Limfjord, a shallow sound that has narrow openings to the Kattegat in the east and the North Sea in the west (1.5 km and 0.5 km wide respectively). Lemvig has areas of low elevation and is in close proximity to water, resulting in flooding due to storm surges on a number of occasions, overflow of sewers in cloud burst events, flooding from increased storm intensity, groundwater flooding, and flooding along streams (Lemvig Kommune, 2014). Within this case study, the coastal hazards Lemvig is and will be exposed to will be described in further detail, as well as the current management approach that has been implemented to address these hazards.

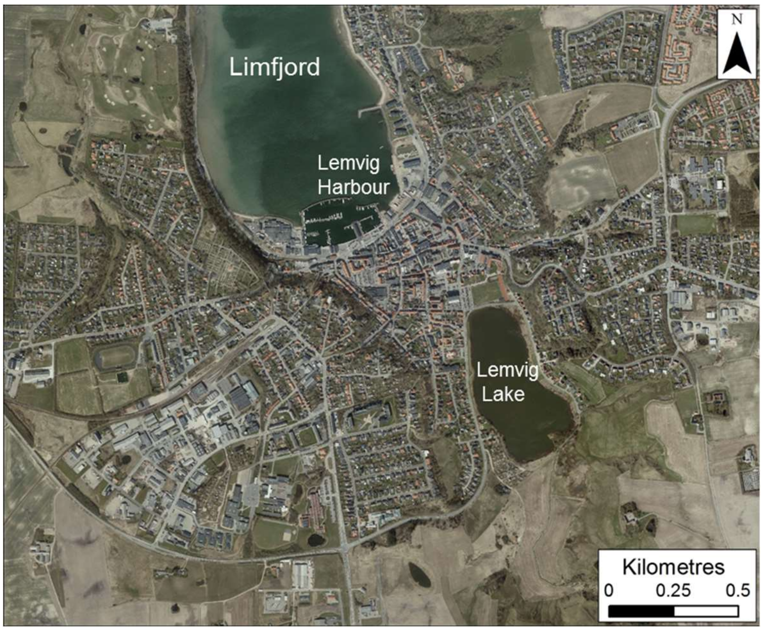

Figure 2: The town of Lemvig, and the location of the two main water sources, the Limfjord and Lemvig Lake.

2.0 Coastal Hazards in Lemvig

Lemvig is situated at the head of narrow inlet of the Limfjord, which makes the town especially prone to storms surges. Further contributing to this risk is climate change with, the mean sea water level in Denmark expected to rise by between 0.3 m and 0.6 m by 2100 (Miljøstyrelsen, 2015). Consequently, this affects the level and return period of storm surge events. In Lemvig, the 10-year storm surge (0.1 probability) is 166 cm, whereas by 2120, this is expected to be 260 cm (**Table 1** and Figure 3).

Table 1: Change in storm surge height with sea level rise in Lemvig *(COWI, 2017)*

Return Period		Estimated surge height for a northerly storm (cm)			
(probability)	(year)	2015	2065	2090	2120
0.1	10	166	203	226	260
0.05	20	173	210	233	267
0.02	50	181	218	241	275
0.01	100	185	222	245	279
0.004	250	191	228	251	285
0.002	500	195	232	255	289
0.001	1000	199	236	259	293
0.0005	2000	202	239	262	296

Therefore, flooding from storm surges is likely to become more severe and more frequent in the future. Within Lemvig, this equates to a storm surge flooding risk of 3.7 million Danish Kroner (approximately $565,000 US Dollars) in 2017, to 440 million Danish Kroner ($67 million US Dollars) in 2117 (COWI, 2017). In addition to storm surges, flooding is also possible via the watercourse that drains Lemvig Lake (Figure 2), just south of the town (Lemvig Kommune, 2014). This can potentially create an 'in combination' risk, as with a high storm surge, the water is not able to drain (via gravity) into the harbour as it usually would.

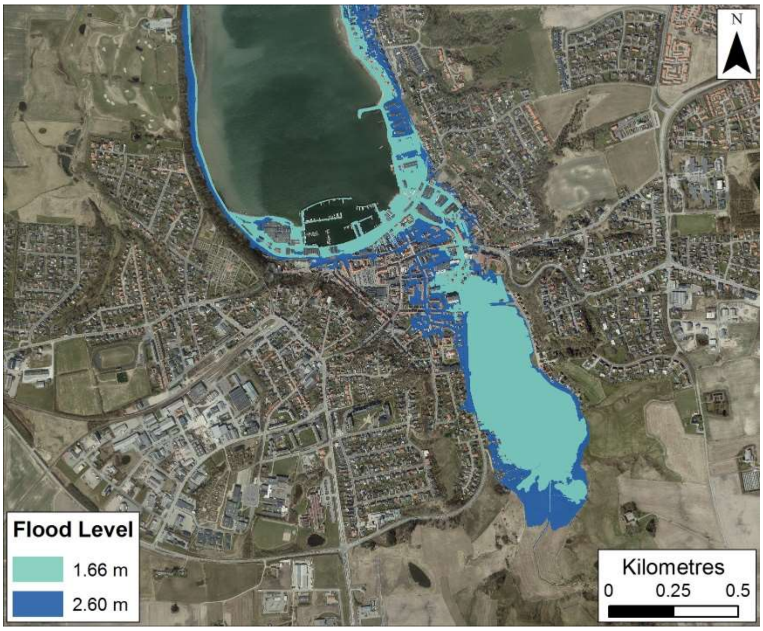

Figure 3: Flood envelopes for Lemvig, Denmark, for the 10-year (0.1 probability) flood in 2017 (1.66 m) and 2117 (2.60 m).

3.0 Flood Management in Lemvig

In response to the coastal flood risk in Lemvig the municipality chose to install a sea wall along the harbour front, as well as redesign the waterfront layout in in 2012-13. The sea wall was designed to be multifunctional, and has a number of gates within it (Figure 4), which are open during normal conditions and allow the public to walk around and utilise the area to sit, as well as access a children's play area (Figure 5)(Faragò, Rasmussen, Fryd, Nielsen, & Arnbjerg-Nielsen, 2018). The gates are closed when a storm surge is predicated therefore the sea wall acts like a traditional barrier. Although this is a hard solution, and could be seen more of as mitigation, rather than adaptation, it demonstrates a mixed use and benefits beyond solely coastal protection.

The sea wall is designed to prevent flooding that is 2.1 m above sea level, and since its construction, a number of storm surges have occurred, with the seawall successfully preventing flooding in the town. This therefore limits the life of the sea wall, as with sea level rise, storm surges and wave overtopping are expected to regularly exceed this height. Initially, the sea wall was expected to give protection for approximately 25 years, with sea wall being redesigned or upgraded in the future (Harbour, 2015). This of course will come at an economical cost, but also if the sea wall is raised further it will being to impinge on the sea view, and the aesthetical quality of the harbour front could be lost. The multifunction of the current sea wall design may not be possible within future designs.

In order to mitigate the flooding created as a result of drainage from Lemvig Lake during a storm surge event a pumping station behind the sea wall at the harbour has been considered (Lemvig Kommune, 2014). The same system could also be used to remove flood water as a result of wave overtopping. As of January 2019 the pumping station has not been implemented.

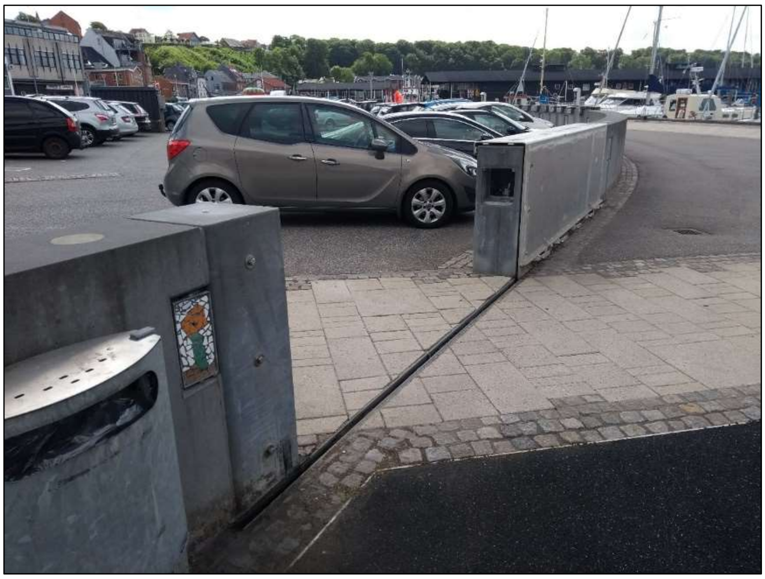

Figure 4: An example of the sea wall in Lemvig with gates that allow pedestrians to use the waterfront, but can be closed during a storm surge event. Photo credit: Authors.

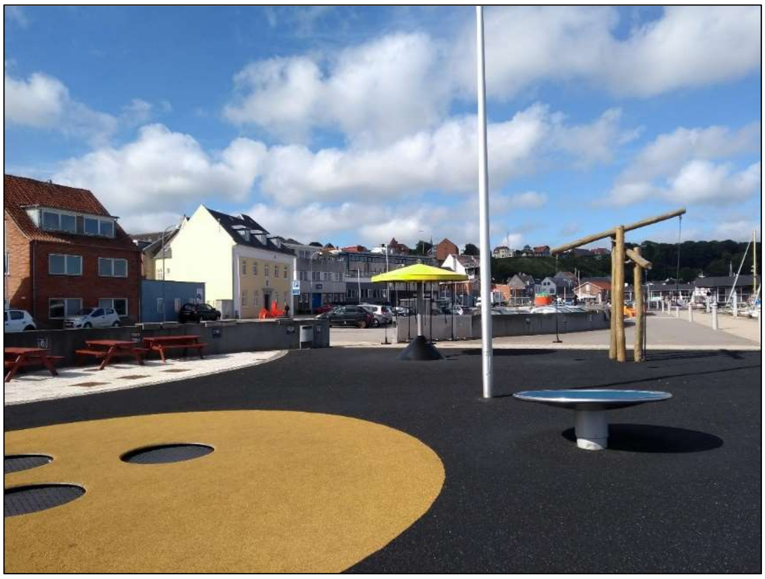

Figure 5: An example of the play area situated in front of the sea wall in Lemvig that would temporarily be flooded during a storm surge event. Photo credit: Authors.

Lemvig is part of the Coast to Coast Climate Challenge (C2C CC) which is a 6 year project (2017 to 2022) focussing on climate change adaptation, and includes municipalities in the Central Denmark Region and 50 partners. The aims of the project are to develop a long-term strategy to adaptation, implementing climate adaptation plans, and identifying and improving resources and capacities among citizens and municipalities, utilities and companies in the water trade (http://www.c2ccc.eu/english/).

As part of the C2C CC, the Climatorium is being built in Lemvig. The Climatorium is a new building that will be built in Lemvig Harbour with the aim of fostering innovation, gathering knowledge on climate change adaptation with regards the sea, coast, and water, and also act as a tourist attraction for the town (http://www.c2ccc.eu/english/sub-projects/action-c21-klimatorium).

4.0 Conclusion

Lemvig is a small town that has been impacted by coastal flooding in the past and is likely to be further at risk due to sea level rise. While currently an innovative multifunctional sea defence structure is in place, this only has a design life of approximately 25 years. Planning for the future is therefore required, and the C2C CC and the Climatorium projects will support this as the town will be able to obtain expertise and knowledge from a range of stakeholders in Denmark to support the adaptation of the harbour front.

5.0 References

COWI. (2017). *RealDania: Byernes udfordringer med havvandsstigning og stormflod*.

Faragò, M., Rasmussen, E. S., Fryd, O., Nielsen, E. R., & Arnbjerg-Nielsen, N. (2018). Coastal protection technologies in a Danish context. Vand i Byer – Innovationsnetværk for klimatilpasning, Taastrup, Denmark., (September).

Harbour, L. (2015). Concrete wall kept the record- high water levels out of Lemvig, 1–3. Retrieved from https://ing.dk/artikel/betonmur-holdt-rekordhoej-vandstand-ude-af-lemvig-173429

Kappel, V., Rasmussen, T., & Waneck, J. (2010). *Danmarks Kyster*. Politikens forlag, Copenhagen.

Lemvig Kommune. (2014). *Klimatilpasningsplan 2014 - 2017*.

Lemvig Kommune. (2017). Datakatalog 2017. Retrieved from https://www.lemvig.dk/Files/Files/Om Kommunen/datakatalog/Datakatalog 2017 - v2.pdf

Miljøstyrelsen. (2015). Ændringer i havniveau.

Sørenson, P. (2013). Denmark. In E. Pranzini & A. Williams (Eds.), *Coastal Erosion and Protection in Europe*. Routledge, London, UK.

Lemvig, Denmark

Table 1: Typology to assess the hazards and susceptibility of a coastal locality

#	Hazard and Susceptibility Elements	Indicators / Metrics	Sources
1	**Settlement location**	North west coast of Jutland, Denmark: 56.55N, 8.30E	
2	**Köppen–Geiger climate classification system**	Currently Cfb - Temperate, without dry season, and warm summers, and expected to remain as such till at least 2100	- Rubel, F., and M. Kottek, 2010: Observed and projected climate shifts 1901-2100 depicted by world maps of the Köppen-Geiger climate classification. Meteorol. Z., 19, 135-141. DOI: 10.1127/0941-2948/2010/0430.
3	**Isostatic rebound**	A value of 0.6 mm/yr of uplift, with a modelled estimates of between 0 and 0.2 m of uplift by 2100	- Sorensen, C., Broge, N.H., Molgaard, M.R., Schow, C.S., Thomsen, P., Vognsen, K., Knudsen, P., 2016. Assessing Future Flood Hazards for Adaptation Planning in a Northern European Coastal Community. Front. Mar. Sci. 3, 69. https://doi.org/10.3389/fmars.2016.00069<br>- Grinsted, A., 2015. Second Assessment of Climate Change for the Baltic Sea Basin, Regional Climate Studies. Springer International Publishing, Cham. https://doi.org/10.1007/978-3-319-16006-1
4	**Subsidence**	10 mm/yr noted in Thyboron (a town 23 km north of Lemvig), but no evidence available for Lemvig	- Sorensen, C., Broge, N.H., Molgaard, M.R., Schow, C.S., Thomsen, P., Vognsen, K., Knudsen, P., 2016. Assessing Future Flood Hazards for Adaptation Planning in a Northern European Coastal Community. Front. Mar. Sci. 3, 69. https://doi.org/10.3389/fmars.2016.00069
5	**Local/regional mass density changes**	Between 0.4 and 0.5 m of sea level rise by 2100	- Grinsted, A., 2015. Second Assessment of Climate Change for the Baltic Sea Basin, Regional Climate Studies. Springer International Publishing, Cham. https://doi.org/10.1007/978-3-319-16006-1
6	**Coastal erosion**	Coastal erosion is not an issue with the town, however within the region coastal erosion is an issue	- Kystdirektoratets Kystatlas http://kms.maps.arcgis.com/apps/webappviewer/index.html?id=8669133b3f4842b7a9a19fb24b08ffd5
7	**Slopes and angles on or near the shore**	Low areas (< 5 m) of land close to shore and inland, with high areas of ca. 25 m in the south west	- Miljøministeriet Kort & Matrikelstyrelsen, 2014. Danmarks Højdemodel 2007 , DHM-2007/Terræn
8	**Located in tropical or other storm zone**	No	- Local knowledge
9	**Inland Rainfall**	Increase in rainfall in winter, spring, and autumn by 30%, 27%, and 9% respectively, with a decrease of 2% in summer in 2081-2100 compared to 1986-2005 under a RCP8.5 scenario	- Miljø- og Fødevareministeriet, n.d. Climate Change Adaptation [WWW Document]. URL https://en.klimatilpasning.dk/maps/
10	**Inland rivers**	Lemvig Lake is situated just south of the town center with an outflow via the harbour. A pumping station is considered in order to aid drainage during storm surges in the Limfjord.	- Lemvig Kommune, 2014. Klimatilpasningsplan 2014 - 2017.
11	**Extent and likelihood of coastal and/or fluvial flooding**	Increase in likihood of higher storm surgesw with 10-year storm surge (0.1 probability) is 166 cm, whereas by 2120, this is expected to be 260 cm.	- COWI, 2017. RealDania: Byernes udfordringer med havvandsstigning og stormflod.
12	**Air temperature**	2.8 to 3.7°C higher in 2081-2100 compared to 1986-2005 under a RCP8.5 scenario	- Miljø- og Fødevareministeriet, n.d. Climate Change Adaptation [WWW Document]. URL https://en.klimatilpasning.dk/maps/
13	**Ocean/Coastal Parameters**	Not available	
14	**Habitats**	None	
15	**Groundwater salinization**	Slight increase of groundwater elevation of between 0 to 1 m by 2050	- Miljø- og Fødevareministeriet, n.d. Climate Change Adaptation [WWW Document]. URL https://en.klimatilpasning.dk/maps/
16	**Base Rock**	Surface lithology: Boulder clay<br>Bedrock: Chalk<br>Bedrock Depth: -870	- Geological Survey of Denmark and Greenland, n.d. Maps of Denmark [WWW Document]. URL https://eng.geus.dk/products-services-facilities/data-and-maps/maps-of-denmark/
17	**Other non-coastal natural hazards**	No other hazards noted	- Local knowledge

Lemvig, Denmark

Table 2: Typology to assess exposure and vulnerability of a coastal locality

#	Exposure and Vulnerability Elements	Indicators / Metrics	Sources
18	Population	6,978	- Danmark Statistiks (2018) Population 1. January by population, area and population density, urban and rural areas and time
19	Future Population Change	Overall -4% from 2017 to 2021, with more than 50% of the residents are aged over 40. While the overall population growth is negative, the predictions for population growth amongst those age 65 and above are increasing	- Lemvig Kommune, 2017. Datakatalog 2017 [WWW Document]. URL https://www.lemvig.dk/Files/Files/Om Kommunen/datakatalog/Datakatalog 2017 - v2.pdf
20	Historic coastal and/or fluvial flooding	Storm surge in 2012 which did no breach sea wall. Numerous coastal flooding events in the past.	- Østergaard, C., 2015. Betonmur holdt rekordhøj vandstand ude af Lemvig. Ingeniøren.
21	Human Development Index (national)	0.929, ranked 11th globally.	- UNDP (2018) Denmark [WWW Document]. URL: http://hdr.undp.org/sites/all/themes/hdr_theme/country-notes/DNK.pdf
22	GNP/capita (probably national)	GDP $54,337 USD per capita	- OECD (2018) Denmark. [WWW Document}. URL: https://data.oecd.org/denmark.htm
23	Proportion of national population that is coastal	40% of the Danish population lives within 3 km of the coast	- Sørenson, P. (2013) 'Denmark', in Pranzini, E. and Williams, A. (eds) Coastal Erosion and Protection in Europe. Routledge, London, UK.
24	Governance	Parliamentary representative democracy, constitutional monarchy and decentralized unitary state	- Local knowledge
25	Relationships to larger governmental entities	Part of Lemvig Municipality (Kommune), which is a part of the Central Denmark region (Midtjylland)	- Local knowledge
26	Relationships to international entities	Lemvig is part of the Coast to Coast Climate Challenge (C2C CC) which is a 6 year project (2017 to 2022) focussing on climate change adaptation, and includes municipalities in the Central Denmark Region and 50 partners (http://www.c2ccc.eu/english/)	- Local knowledge
27	Built Infrastructure	At the immedied coast there is a harbour with industrial and recreational assets, with a key road and residential properties near the coast. The newly built Climatorium is also located at the coast.	- Local knowledge
28	Natural Capital	Not applicable	
29	Available geographic/GIS data	Signficiant amounts of data available that is regularly updated. This is the responsibility of the national mapping agency to maintain. Climate modelling at national scales is also available	- Local knowledge
30	Minority status	None	- Local knowledge
31	Historical areas	A number of historic buildings within the town e.g. Lemvig Church	- Local knowledge
32	Environmental areas	None in the immediate vicinity of the town	- Local knowledge
33	Cultural areas		
34	Tourism areas	Some tourism to the town but relativly minor. The development of the Climatorium will potentially increase tourism to the town	- Local knowledge
