# Supplementary material for: Challenges to climate change adaptation in coastal small towns: Examples from Ghana, Uruguay, Finland, Denmark, and Alaska
Source: Ocean Coast Manag. 2021 Oct 15;212:105787. doi: 10.1016/j.ocecoaman.2021.105787 (PMC10644629; doi:10.1016/j.ocecoaman.2021.105787)
Supplement: Multimedia component 5 [file mmc5.pdf]

Climate Change Stressors and Reponses in Nome, Alaska

S. Jeff Birchall[1*]

[1]School of Urban and Regional Planning, University of Alberta

Abstract

Climate change can facilitate both risk and opportunity. In Nome, Alaska for instance, improved access to natural resources and tourism can bolster the local economy, and thus improve quality of life for those living in there. However, when change occurs rapidly and impacts are extreme and variable, the infrastructure and policies necessary to support new opportunities can be challenged. Resilience in small coastal towns can be further hindered by a lack of resources and political consensus. In Nome, thawing permafrost and variable sea ice are the two key stressors influencing the community. While decision-makers in Nome accept that these stressors will worsen as the climate continues to warm, adaptation remains a low priority.

1.0 Location and economy

Nome is a small community situated in the Nome Census Area, on the southern coast of the Seward Peninsula, in northwest Alaska (Figure 1). The community of Nome is remote from major urban centres in the interior such as Anchorage and Fairbanks, with access limited to sea and air (Anchorage: 870km; Fairbanks: 840km) (City of Nome, 2012). Local access to outlying communities such as Council (to the east) and Teller (to the west) is restricted to seasonal roads.

Incorporated in 1901, Nome was once a bustling gold mining town with a population of over 20,000 residence (City of Nome, 2017). Since then, Nome's population has decreased significantly, primarily as a result of a decline in the gold industry, disease outbreak (i.e. Spanish Flu) and war. With a current population of 3500[2], Nome remains the largest community in the region, and serves as a hub for the provision of health and education, and commercial and industrial activities. Locally, employment is dominated by education and health care (27%), government (26%), and trades, transportation and utilities (22%) (City of Nome, 2012).

---

*Corresponding Author: jeff.birchall@ualberta.ca

[2] http://www.visitnomealaska.com/history-culture/

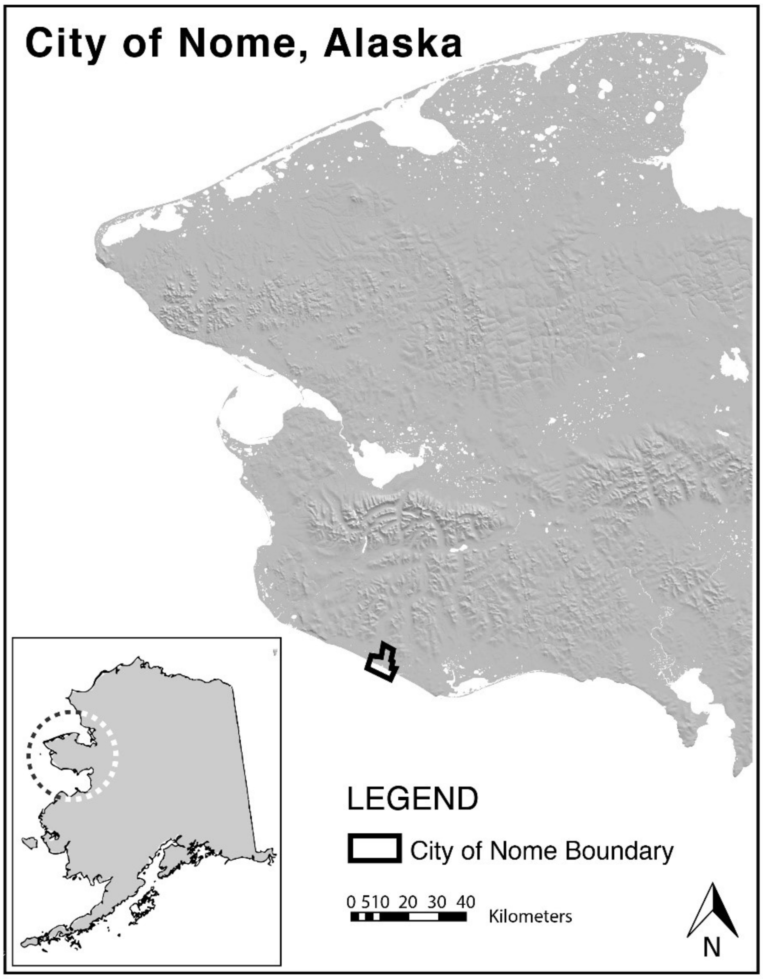

Figure 1: The City of Nome, Alaska. The following is the source information used by the author to create the map: Coordinate System (Birchall and Bonnett, 2020): NAD 1983 Alaska Albers; Projection: Albers; Datum: North American 1983; Data Source: Alaska Department of Natural Resources State Geo-Spatial Data Clearinghouse; Date Created: June 25, 2018.

A key feature of Nome's landscape is the port, which sits at the mouth of the Snake River. The port is the sole provider of moorage and services in the region, and can accommodate bulk cargo and fuel (City of Nome, 2012). Recently the US Army Corps of Engineers and the City of Nome agreed to commission a study to assess the value of expanding the port so that it can allow deep-draft vessels (Grueskin, 2018). The expansion of the port is seen as an opportunity to diversify the State's economy, which is largely centred around oil and gas extraction (Grueskin, 2018). Further, with a longer open water season, an expanded port would not only allow for more commercial cargo activity, it would also facilitate local economic growth through increased cruise ship and pleasure craft traffic.

2.0 Environment and climate

Nome sits in a coastal plain (low elevation), with Anvil Mountain to the north and the Bering Strait to the west; nearly half the community is in a flood plain (City of Nome, 2017). The landscape is defined by tundra (there are no trees!), and Nome is underlain by perennially frozen ground (City of Nome, 2017). Relative to much of the State, the permafrost layer beneath the community is thin, at approximately 15-50 meters (Hinzman et al., 2005).

Nome's climate is classified as subarctic, and characterized by short cool summers and long cold winters (City of Nome, 2012). July is the warmest month with an average temperature of 11C, February the coolest, with an average temperature of -14C; average annual temperature is -2.8C (City of Nome, 2012). With respect to precipitation, March is the driest month, and August the wettest; snowfall averages 145 cm over the year (City of Nome, 2012).

The community is susceptible to severe weather, including freezing rain, winter storms and ocean surge, which can result in flooding and erosion (City of Nome, 2017).

3.0 Climate change

Over the past 60 years, Alaska has seen temperatures rise at a pace 2x the rest of the United States (e.g. Stewart et al., 2013). In Nome, mean seasonal temperature increased approximately 2C between 1949-2016 (unit converted from ACRC, 2017).

Towards the end of this century, temperatures across Alaska are projected to increase by 2.4-6.3C, and precipitation by 14-28% by 2100 (Christensen et al., 2007). These trends are echoed across the Arctic in general, where simulations reveal an increase in temperature in the order of 3-6C by 2080, and a rise in precipitation through 2100, with a significant increase relative to global average (AMAP, 2012).

3.1. Key climate change stressors

Climate change impacts are not a new risk in Alaska, however they have begun to increase in occurrence and magnitude (ADEC, 2010; Melvin et al., 2016). In Nome, rising temperatures present two principal stressors: thawing permafrost and variable sea ice.

3.1.1. Thawing permafrost:

As temperatures rise in Nome, the permafrost layer beneath the community is also warming. Across the State, data indicate that permafrost temperatures have increased by 2C over the last 30 years (Alaska Sea Grant, 2018a). In Nome, this results in permafrost becoming increasingly

thaw unstable as the active layer extends deeper into the perennially frozen ground (City of Nome, 2017). This challenges infrastructure in three main ways (Birchall and Bonnett, 2020):

3.1.2. Building subsidence

Structures built on permafrost need to be designed in such a way that heat can escape up and out, rather than transfer into the ground where it will thaw the permafrost and destabilize the structure above (ADEC, 2010). In Nome, buildings constructed on permafrost rest on meter-high stilts, piles or stacks of blocks, which allows the building's heat to dissipate. Historically, this design would limit the need for levelling, however because of the warmer ambient air temperature, levelling now required annually (Birchall and Bonnett, 2020). Larger structures such as the Norton Sound Regional Hospital run freon through the piles to help keep the ground cool and thus stable.

3.1.3. Utilities failure

Permafrost thaw also influences water and waste water pipes. During the warmer months, the unstable ground leads to mainlines heaving, dropping and separating throughout the looped system (Birchall and Bonnett, 2020). While utilities crews effort to mitigate risk by excavating deeper into thaw stable ground, service disruption is occurring more often, with environmental risk and erosion on the rise (Birchall and Bonnett, 2020).

3.1.4. Road undulation

In high latitudes, surfaces with minimal reflectivity (low albedo), such as roads, absorb a great deal of heat during the summer month period when sunlight persists for extended periods of time. This heat is transferred, in part, to the ground below, which can destabilize the permafrost layer and cause the paved surface above to heave and/ or undulate. In Nome, this condition translates into consistent infrastructure repair, with infrastructure lifespan typically reaching about 4 years. With the addition of warmer ambient temperatures, infrastructure lifespan has been reduced to 1 year. In order to mitigate risk and reduce maintenance costs, much of Nome's road network consists of dirt/ gravel surfaces (Birchall and Bonnett, 2020).

3.1.5. Variable sea ice

Warmer temperatures have resulted in a decline in Arctic sea ice (IPCC, 2014). Projections for the south Bering Strait suggest that by 2050 winter-month sea ice cover could be fifty per cent the average of recent years (e.g. Overland and Wang, 2007). Weaker winter ice cover translates

into thinner summer ice; the lowest summer sea ice extents have occurred in the last 10 years (Stroeve et al., 2017).

Sea ice can serve to buffer the force of winter storms. However, sea ice in the Bering Strait is thawing earlier in spring and forming later in the winter season, which results in a longer open water period (e.g. Stroeve et al., 2012). Without a sea ice cover a larger ocean fetch is possible (e.g. Wadhams, 2012), which allows for larger surge. Moreover, weaker sea ice, and in particular, weaker shore-fast ice is less effective at dissipating wave energy and reducing wave contact with the coastline (Overeem et al., 2011; Alaska Sea Grant, 2018b). In combination with stronger storms and surge, this can result in greater flooding and erosion along Nome's coast (City of Nome, 2017).

Indeed, fall/ winter storm are stressing the seawall that protects downtown Nome, as evidenced by slumping of the armour stone (Birchall and Bonnett, 2020*,* and sections of town near the coast are becoming increasingly susceptible to flooding (FEMA, 2010). Initially built by the federal government, ongoing sea wall maintenance is the responsibility of Nome (City of Nome, 2012). The Nome-Council Highway is likewise suffering from erosion, which has resulted in road collapse.

4.0 Policy response

Decision-makers in Nome accept that stressors like thawing permafrost and sea ice variability will continue to necessitate attention moving forward, especially in the context of infrastructure. With that said, officials characterize their efforts as reactionary, rather than anticipatory (Birchall and Bonnett, 2020). The lack of forward movement on climate change adaptation may be the result of it's low priority, as demonstrated by the community's strategic planning documents and zoning policy.

The Nome Comprehensive Plan 2020, for example, classes climate change adaptation action as a long-term strategy, with initiation not expect within next 5 years; even then, focus is on developing infrastructure to support the increase in marine traffic (City of Nome, 2012).

The Hazard Mitigation Plan, a requirement under the Federal Emergency Management Agency, while highlighting the increasing risk of flooding and erosion, is similarly lite on commitment to adaptation. For instance, the most recent plan (2017) does not carry forward key actions from the previous plan (2008) which if implemented, could go some way to improving resilience (City of Nome, 2017):

- establish zoning code and subdivision regulations that are specific to development within the flood prone areas
- limit building city owned buildings in the 100 and 500-year flood zone
- obtain funding to increase the height and impermeability of the Nome seawall

The plan does carry forward some important action, however (City of Nome, 2017):

- determine vulnerability of flood prone buildings
- improve access to flood insurance
- improve community awareness about flood risk in general

Importantly, the recent plan also includes an action to understand how to effectively build on thawing permafrost (City of Nome, 2017).

The community's Emergency Operations Plan also includes language around flood proofing and evacuation, as well as information relating the importance of back-up resources in the event of extreme weather (remote location, easily cut off from supplies) (City of Nome, 2011).

At a more fundamental scale, the Flood Hazard Overlay district of the Nome Zoning Code, also may not go far enough to foster resilience. For instance, the district does not set a higher level for setbacks, or limit the allowable use within the zone (City of Nome, 2008). It does however require that flood risk be mitigated through an Elevation Certificate and a Flood Plain Permit (which requires details about the site and evidence of flood proofing) (City of Nome, 2008), obtained through the National Flood Insurance Program and the City of Nome, respectively.

Policy action in Nome is further challenged by a distinct lack of funding, as identified by the Nome Comprehensive Plan 2020 (City of Nome, 2012) and the Emergency Operations Plan (City of Nome, 2011). While this is primarily the result of a small tax base, fiscal constraints at the State level contribute to the difficulty (e.g. Flavelle, 2017). While this makes infrastructure maintenance difficult, it also influences the community's ability to solicit experts to train key staff on how to incorporate climate change adaptation into existing strategic policy (Birchall and Bonnett, 2020).

At the State level, in 2010, the Climate Change Sub-cabinet released *Alaska's Climate Change Strategy* (ADEC 2010), and in so doing demonstrated their interest in working with local government on this matter. Highlighting risk in the context of public infrastructure, natural systems, economic, health and cultural concerns, the document serves as a resource to help decision-makers appreciate how climate change may impact their communities and plots the State's path forward.

In 2011, Alaska Governor Parnell shut-down the Sub-cabinet, and in the following years the Department of Environmental Conservation had its budget significantly reduced (Flavelle 2017). More recently, Governor Walker struck a climate action leadership team to explore how best to move forward on the climate change agenda (Evengaard, 2018).

However, in a State where the economy is tightly linked to oil extraction, and with the head of the US Environmental Protection Agency arguing against the link between rising atmospheric greenhouse gas emissions and rising global temperatures (Milman, 2017), funding for climate change action, whether for mitigation or adaptation, may remain a challenge for local government's like Nome.

5.0 Conclusion

Nome, Alaska is a small coastal town experiencing the impacts of the warming climate. While climate change is not new to communities in the Arctic, impacts have begun to increase in occurrence and magnitude. In Nome, thawing permafrost leads to building subsidence, utilities failure and road undulation; and, variable sea ice can affect storm surge and influence how wave energy is dissipated along the seawall. While decision-makers in Nome understand the impacts will continue to stress the community, adaptation remains a low priority within strategic planning documents and zoning policy.

6.0 References

ACRC. (2017). Temperature Changes in Alaska. Alaska Climate Research Centre, University of Alaska, Fairbanks. Accessed October 22, 2017: http://akclimate.org/ClimTrends/Change/TempChange.html.

ADEC. (2010). Alaska's Climate Change Strategy: Addressing Impacts in Alaska. Alaska Department of Environmental Conservation. Accessed July 24, 2018: http://www.adaptationclearinghouse.org/resources/alaska-s-climate-change-strategy-addressing-impacts-in-alaska.html.

Alaska Sea Grant. (2018a). Permafrost change: What it means to Alaskans and how we can adapt. Living on Alaska's changing coasts. Accessed June 27, 2018: https://seagrant.uaf.edu/bookstore/pubs/M-144.html.

Alaska Sea Grant. (2018b). Sea level rise and storm surge: What it means to Alaskans and how we can adapt. Living on Alaska's changing coasts. Accessed June 27, 2018: https://seagrant.uaf.edu/bookstore/pubs/M-144.html.

AMAP. (2012). Arctic Climate Issues 2011: Changes in Arctic Snow, Water, Ice and Permafrost. Snow, Water, Ice and Permafrost in the Arctic (SWIPA) 2011 Overview Report, Arctic Monitoring and Assessment Programme.

Birchall, S.J., Bonnett, N. (2020) Thinning sea ice and thawing permafrost: climate change adaptation planning in Nome, Alaska, Environmental Hazards, 19:2, 152-170, DOI: 10.1080/17477891.2019.1637331

Christensen, J.H., Hewitson B., Busuioc A., et al. (2007) Regional climate projections. In: Climate Change 2007: The Physical Science Basis. Contribution of Working Group I to the Fourth Assessment Report of the Intergovernmental Panel on Climate Change (eds Solomon S, Qin D, Manning M, et al.), pp. 847–940. Cambridge University Press, Cambridge, UK and New York, NY, USA

City of Nome. (2017). City of Nome, Alaska Hazard Mitigation Plan Update. City of Nome, Alaska. Accessed July 18, 2018: https://www.nomealaska.org/egov/documents/1491941425_32199.pdf.

City of Nome. (2011). Emergency Operations Plan, Nome, Alaska. City of Nome, Alaska. Accessed July 19, 2018: https://www.nomealaska.org/egov/documents/1368224192_422668.pdf.

City of Nome. (2012). Nome Comprehensive Plan 2020. City of Nome, Alaska. Accessed July 19, 2018: https://www.nomealaska.org/egov/documents/1349978949_808227.pdf.

City of Nome. (2008). Nome Zoning Code. City of Nome, Alaska. Accessed Oct 29, 2018: https://www.nomealaska.org/egov/documents/1349907967_667724.pdf.

Evengaard, A. (2018). Alaska Governor issues order on climate change strategy. GlacierHub. Accessed: October 30, 2018: http://glacierhub.org/2018/01/09/alaska-governor-issues-order-climate-change-strategy/.

FEMA. (2010). Flood Insurance Study - City of Nome, Alaska. Federal Emergency Management Agency. Accessed July 23, 2018: https://www.nomealaska.org/egov/documents/1304705573_125335.pdf.

Flavelle, C. (2017). Alaska's big problem with warmer winters. Bloomberg. Accessed May 16, 2018: https://www.bloomberg.com/news/articles/2017-03-09/alaska-s-big-problem-with-warmer-winters.

Grueskin, Z. (2018). Nome deep-draft port back on the table. Alaska Public Media. Accessed October 26, 2018: https://alaskapublic.org/2018/02/08/nome-deep-draft-port-back-on-the-table/.

Hinzman, L.D., Bettez, N. D., Bolton, W. R., Chapin, F. S., Dyurgerov, M. B, Fastie, C.L., et al. (2005). Evidence and implications of recent climate change in Northern Alaska and other Arctic regions. Climatic Change, 72, 251-298.

IPCC. (2014). Synthesis Report. In: Climate Change 2014. Contribution of Working Group I, II and III to the Fourth Assessment Report of the Intergovernmental Panel on Climate Change, edited by Pachauri, R. K., and L. A. Meyers. Geneva, Switzerland: IPCC.

Melvin, A. M., Larsen, P., Boehlert, B., Neumann, J. E., Chinowsky, P., Espinet, X., et al. (2016). Climate change damages to Alaska public infrastructure and the economics of proactive action. PNAS, E122-E131. www.pnas.org/cgi/doi/10.1073/pnas.1611056113.

Milman, O. (2017). EPA head Scott Pruitt denies that carbon dioxide causes global warming. The Guardian. Accessed: October 30, 2018: https://www.theguardian.com/environment/2017/mar/09/epa-scott-pruitt-carbon-dioxide-global-warming-climate-change.

Overeem, I., Anderson, R., Wobus, C., Clow, G., Urban, F. & Matell, N. (2011). Sea ice loss enhances wave action at the Arctic coast. Geophysical Research Letters, 38. DOI: 10.1029/2011GL048681.

Overland, J. E. & Wang, M. (2007). Future regional Arctic sea ice declines. Geophysical Research Letters, 34. doi:10.1029/2007GL030808.

Stewart, B. C., K. E. Kunkel, L. E. Stevens, L. Sun, and J. E. Walsh. (2013). Regional Climate Trends and Scenarios for the U.S. National Climate Assessment: Part 7. Climate of Alaska. NOAA Technical Report NESDIS 142-7.

Stroeve, J. C., Kattsov, V., Barrett, A., Serreze, M., Pavlova, T. Holland, M., and Meier, W. N. (2012). Trends in Arctic sea ice extent from CMIP5, CMIP3 and observations. Geophysical Research Letters. https://doi.org/10.1029/2012GL052676.

Stroeve, J., Serreze, M., Ornaheim, I., Holland, M., Vavrus, S., Meier, W., and Fetterer, F. (2017). Arctic sea ice in 2016: A preview of the future. In EGU General Assembly Conference Abstracts, 19, 6110.

Wadhams, P. (2012). Arctic ice cover, ice thickness and tipping points. Ambio, 41(1), 23-33. doi: 10.1007/s13280-011-0222-9.

Nome, Alaska, USA

Table 1: Typology to assess the hazards and susceptibility of a coastal locality

#	Hazard and Susceptibility Elements	Indicators / Metrics	Sources
1	**Settlement location**	Latitude: 64.5011 N; Longitude: 165.4064 W;<br>Total corporate boundaries include 32km2 of land, 23.6km2 of water;<br>6 m above sea level	- City of Nome. (2017). City of Nome, Alaska Hazard Mitigation Plan Update. City of Nome, Alaska. Accessed July 18, 2018: https://www.nomealaska.org/egov/documents/1491941425_32199.pdf.
2	**Köppen–Geiger climate classification system**	sub-arctic climate (long very cold winters; short cool summers)	- City of Nome. (2017). City of Nome, Alaska Hazard Mitigation Plan Update. City of Nome, Alaska. Accessed July 18, 2018: https://www.nomealaska.org/egov/documents/1491941425_32199.pdf.
3	**Isostatic rebound**	no information on relative sea level change in Nome due to data collection time line not yet sufficient	NOAA
4	**Subsidence**		
5	**Local/regional mass density changes**	Limited empirical evidence, but very little relative sea level rise expected	- Church, J.A., Clark, P.U., Cazenave, A., Gregory, J.M., Jevrejeva, S., Levermann, A., Merrifield, M.A., Milne, G.A., Nerem, R.S., Nunn, P.D., Payne, A.J., Pfeffer, W.T., Stammer, D., Unnikrishnan, A.S., 2013. Sea Level Change, in: Stocker, T.F., Qin, D., Plattner, G.-K., Tignor, M., Allen, S.K., Boschung, J., Nauels, A., Xia, Y., Bex, V., Midgley, P.M. (Eds.), Climate Change 2013: The Physical Science Basis. Contribution of Working Group I to the Fifth Assessment Report of the Intergovernmental Panel on Climate Change. Cambridge University Press, Cambridge, United Kingdom and New York, NY, USA.
6	**Coastal erosion**	significant coastal wind erosion (City of Nome, 2017)<br>significant coastal and river erosion, primarily the result of storm surges at high tide and winds from Norton Sound (USACE, 2008). Erosion linked to jetty placement and harbour entrance; erosion along Nome-Council Road, affects road quality<br>Nome waterfront erosion: receded 50-80ft 1904-2010 (City of Nome, 2010)	US Army Corp of Engineers;<br>State Government
7	**Slopes and angles on or near the shore**	less shore-fast ice means greater direct wave contact with the coastline (Overeem et al., 2011; Alaska Sea Grant, 2018b)	- Overeem, I., Anderson, R., Wobus, C., Clow, G., Urban, F. & Matell, N. (2011). Sea ice loss enhances wave action at the Arctic coast. Geophysical Research Letters, 38. DOI: 10.1029/2011GL048681<br>- Alaska Sea Grant. (2018b). Sea level rise and storm surge: What it means to Alaskans and how we can adapt. Living on Alaska's changing coasts. Accessed June 27, 2018: https://seagrant.uaf.edu/bookstore/pubs/M-144.html.
8	**Located in tropical or other storm zone**	Sea ice extent has declined in Arctic Ocean (IPCC, 2014); lowest 10 summer ice extents have all occurred within the last decade (e.g. Stroeve et al. 2017)<br>Projections: by 2050, south of the Bering Strait, winter sea ice cover could be 50% the average of recent years (e.g. Overland and Wang, 2007)<br>Open water days are on the rise (eg. Stroeve et al 2012); larger ocean fetch results in more powerful waves (eg. Wadhams)<br>there are no tide records or wave observation data for Nome coastline (City of Nome, 2012); time line too short	- City of Nome. (2012). Nome Comprehensive Plan 2020. City of Nome, Alaska. Accessed July 19, 2018: https://www.nomealaska.org/egov/documents/1349978949_808227.pdf.<br>- IPCC. (2014). Synthesis Report. In: Climate Change 2014. Contribution of Working Group I, II and III to the Fourth Assessment Report of the Intergovernmental Panel on Climate Change, edited by Pachauri, R. K., and L. A. Meyers. Geneva, Switzerland: IPCC<br>- Overland, J. E. & Wang, M. (2007). Future regional Arctic sea ice declines. Geophysical Research Letters, 34. doi:10.1029/2007GL030808<br>- Stroeve, J. C., Kattsov, V., Barrett, A., Serreze, M., Pavlova, T. Holland, M., and Meier, W. N. (2012). Trends in Arctic sea ice extent from CMIP5, CMIP3 and observations. Geophysical Research Letters. https://doi.org/10.1029/2012GL052676<br>- Stroeve, J., Serreze, M., Ornaheim, I., Holland, M., Vavrus, S., Meier, W., and Fetterer, F. (2017). Arctic sea ice in 2016: A preview of the future. In EGU General Assembly Conference Abstracts, 19, 6110
9	**Inland Rainfall**	Heavy rain is a severe threat as permafrost is less permeable depending on seasonality (winter rains prevent infiltration, increasing flash flood risk);	- Local knowledge
10	**Inland rivers**	Snake River which is susceptible to erosion	US Army Corp of Engineers;<br>local knowledge

11	**Extent and likelihood of coastal and/or fluvial flooding**	experiences significant damage from storm surge (primarily during fall; southeast the most severe), coastal ice-run up<br>Susceptible to erosion, flooding;<br>residential development in floodplain	US Army Corp of Engineers;<br>local knowledge
12	**Air temperature**	July is the warmest month with an average temperature of 11C; February the coolest, with an average temperature of -14C;<br>Average annual temperature is -2.8C (City of Nome, 2012)<br>In Nome, mean seasonal temperature increased approximately 2C between 1949-2016 (ACRC, 2017)<br>Towards the end of this century, temperatures across Alaska are projected to increase by 2.4-6.3C (Christensen et al., 2007)	- US Weather Service
13	**Ocean/Coastal Parameters**	Not available	
14	**Habitats**	Tundra (permafrost thaw)	
15	**Groundwater salinization**		
16	**Base Rock**	Surficial deposits, undivided	- US Geological Survey
17	**Other non-coastal natural hazards**	Earthquakes (periodic, unpredictable); experienced no earthquakes over M5 since 1978;<br>Fire risk from wildland and tundra<br>permafrost degradation; permafrost layer beneath the community is relatively thin, at approximately 15-50 meters , highly susceptible to warming.<br>Across the State, data indicate that permafrost temperatures have increased by 2C over the last 30 years	- City of Nome. (2017). City of Nome, Alaska Hazard Mitigation Plan Update. City of Nome, Alaska. Accessed July 18, 2018: https://www.nomealaska.org/egov/documents/1491941425_32199.pdf.<br>- Hinzman, L.D., Bettez, N. D., Bolton, W. R., Chapin, F. S.,. Dyurgerov, M. B, Fastie, C.L., et al. (2005). Evidence and implications of recent climate change in Northern Alaska and other Arctic regions. Climatic Change, 72, 251-298.<br>- Alaska Sea Grant. (2018a). Permafrost change: What it means to Alaskans and how we can adapt. Living on Alaska’s changing coasts. Accessed June 27, 2018: https://seagrant.uaf.edu/bookstore/pubs/M-144.html.

Nome, Alaska, USA

Table 2: Typology to assess exposure and vulnerability of a coastal locality

#	Exposure and Vulnerability Element	Indicators / Metrics	Sources
18	**Population**	3,598<br>Population declined due to slow-down in good extraction, disease outbreak and war. The current population stable.	US Census Bureau<br>- City of Nome. (2012). Nome Comprehensive Plan 2020. City of Nome, Alaska. Accessed July 19, 2018: https://www.nomealaska.org/egov/documents/1349978949_808227.pdf.
19	**Future Population Change**	1.29%/ yr	- population.us
20	**Historic coastal and/or fluvial flooding**	since 1900 Nome has experienced a number of storm-surge induced floods: 1900, 1902, 1913, 1937, 1942, 1945, 1946, 1972, 1974, 1992, 2004, 2005, 2009 (City of Nome, 2017)	
21	**Human Development Index (national)**	Ranked 13, HDI: 0.924; Life expectancy: 79.5; expected years of schooling: 16.5; although not applicable to remote northern community	- UNDP (2018) United States [WWW Document]. URL: http://hdr.undp.org/sites/all/themes/hdr_theme/country-notes/USA.pdf
22	**GNP/capita (probably national)**	Gross national income per capita: $54,941 with per caput income in Nome: $32,338 (City of Nome, 2012), although not applicable to remote northern community.	- City of Nome. (2012). Nome Comprehensive Plan 2020. City of Nome, Alaska. Accessed July 19, 2018: https://www.nomealaska.org/egov/documents/1349978949_808227.pdf.<br>- UNDP (2018) United States [WWW Document]. URL: http://hdr.undp.org/sites/all/themes/hdr_theme/country-notes/USA.pdf
23	**Proportion of national population that is coastal**		
24	**Governance**	Council/ manager; elected mayor, 6 council members, and rely on professionals from interior (e.g. for planning)	
25	**Relationships to larger governmental entities**	incorporates State and Federal policy into local strategic documents as necessary	
26	**Relationships to international entities**		
27	**Built Infrastructure**	Housing: 1503 units<br>Access to Nome is via air or sea; with over 230 miles of roads connecting w outlying villages.<br>Port and harbour; handles bulk cargo, fuel deliveries, aggregate, recreational and fishing vessels, smaller cargo and landing craft<br>Seawall (rock-riveted slope, 18ft tall)<br>Nome Airport, 2 paved runways (77 operations/ day)<br>Norton Sound Regional Health Corporation Regional Hospital<br>susceptible to permafrost thaw	- City of Nome. (2012). Nome Comprehensive Plan 2020. City of Nome, Alaska. Accessed July 19, 2018: https://www.nomealaska.org/egov/documents/1349978949_808227.pdf.
28	**Natural Capital**	beach/ coastal erosion<br>reduced sea ice<br>permafrost degradation	- Local knowledge
29	**Available geographic/GIS data**	Basic due to very remote location	Alaska Department of Natural Resources State Geo-Spatial Data Clearinghouse
30	**Minority status**	54.8% of population is Alaska Native; 30.4% white (City of Nome, 2012)<br>Nome: regional and economic hub for 16 tribal villages in the region	US Census Bureau
31	**Historical areas**	A busy city during gold rush in 1900, w population over 20,000 (City of Nome, 2017)	- City of Nome. (2017). City of Nome, Alaska Hazard Mitigation Plan Update. City of Nome, Alaska. Accessed July 18, 2018: https://www.nomealaska.org/egov/documents/1491941425_32199.pdf.
32	**Environmental areas**		
33	**Cultural areas**	Subsistence living (coastal fishing); remote community, made difficult in winter due to inconsistent sea ice	- Local knowledge
34	**Tourism areas**	Cruise ship and pleasure craft related tourism on the rise with increase in open-water season;<br>Iditarod dog race, facing challenges as snow is less consistent in timing;<br>increased shipping traffic a concern (environmental)	- Local knowledge
